# Strain-Dependent Network Activity Differences Underlie Social Decision-Making in Male Mice

**DOI:** 10.1523/ENEURO.0207-26.2026

**Published:** 2026-08-21

**Authors:** Elizabeth Illescas-Huerta, Andrea Villamizar, Meghan Cum, Nancy Padilla-Coreano

**Affiliations:** Department of Neuroscience, University of Florida, Gainesville, Florida 32610

**Keywords:** decision-making, mouse strain, network, prefrontal cortex, prosocial behavior

## Abstract

Adaptive social behavior requires balancing self-interest with the welfare of others, a core axiom of social decision-making that determines whether actions are selfish or prosocial. Although the medial prefrontal cortex (mPFC) has been implicated in prosocial behavior, the broader cortical–subcortical networks that arbitrate between selfish and prosocial actions remain poorly understood. Moreover, most studies of social decision-making (at both the single-region and circuit levels) have focused on inbred C57BL/6 mice, leaving unclear whether similar neural mechanisms operate across genetically diverse populations. Here, we combined a social decision-making task with c-Fos mapping to examine activity across distributed cortical and subcortical regions in inbred C57BL/6 and outbred CD1 male mice during prosocial and selfish choices. We found that CD1 mice exhibited a stronger bias toward selfish behavior, whereas C57BL/6 mice were more prosocial. This behavioral divergence was associated with elevated c-Fos activity in the mPFC and nucleus accumbens core (NAcC) in CD1 mice compared with C57BL/6 mice, with mPFC activity positively correlating with selfish choices. At the network level, social decision-making selectively recruited coordinated activity among the distinct mPFC subregions, ventral tegmental area (VTA), and NAcC. Importantly, prosocial and selfish individuals exhibited distinct prefrontal–subcortical network configurations during social decision-making. Together, these findings identify distributed cortical–subcortical network dynamics underlying social choice bias and suggest that differences in the neural architecture associated with prosocial and selfish behavior are influenced by strain in male mice.

## Significance Statement

Social decisions require weighing personal benefit against the welfare of others, yet the neural circuits that bias individuals toward selfish versus prosocial choices remain poorly understood. Here, we show that two mouse strains with opposing social preferences recruit distinct cortical and subcortical network configurations during social choice, despite performing the same task. Rather than reflecting differences in single brain regions, social decision-making engaged coordinated activity across a prefrontal–striatal–midbrain circuit, with prosocial and selfish individuals recruiting different versions of this network. These findings highlight distributed circuit organization as a neural correlate of social choice bias and suggest that genetic background contributes to differences in the neural organization associated with social decision-making.

## Introduction

In social contexts, adaptive behavior depends on the ability to integrate self-interest with the interests of others, a central component of social decision-making (Rilling and Sanfey, 2011). Within this framework, social decisions can be biased toward prosocial choices—defined as intentional actions that confer benefits to others—or selfish actions that prioritize individual gain. Prosocial decisions are conserved across species and play a critical role in maintaining social cohesion and supporting group survival (Rault, 2019; Wu and Hong, 2022). In rodents, prosocial behaviors such as cooperation, harm avoidance, helping, and reward provision have been widely used to investigate the neural basis of social decision-making (Keysers et al., 2022; Gachomba et al., 2024). In contrast, the neural mechanisms that bias decisions toward selfish outcomes, including the brain's mechanisms for arbitrating between prosocial and selfish actions, remain largely unknown.

The medial prefrontal cortex (mPFC) has emerged as a central hub for social decision-making (Gangopadhyay et al., 2021), positioning it as a key region for arbitrating between prosocial and selfish actions. Within the mPFC, distinct subregions support different expressions of prosocial behavior in mice. Activity in the anterior cingulate cortex (ACC) is necessary for harm avoidance toward conspecifics (Song et al., 2023) and plays a role in empathy-like behavior (Smith et al., 2021), whereas the prelimbic cortex (PL) is engaged during cooperation for mutual rewards (Conde-Moro et al., 2024). On the other hand, the role of the infralimbic (IL) subregion in prosocial behavior has not been investigated. In addition, disrupting other cortical areas, such as the insular cortex (IC), decreased prosocial actions, including helping (Cox et al., 2022) and interacting with vulnerable conspecifics (Rogers-Carter et al., 2018), highlighting that prosocial decisions depend on coordinated activity across multiple cortical regions.

Consistent with this view, emerging evidence indicates that social decision-making also relies on interactions between cortical and subcortical circuits. For example, communication between PL and the basolateral amygdala (BLA) is required to share a reward with a partner (Scheggia et al., 2022), ACC–mediodorsal thalamus (MD) connectivity supports harm avoidance toward conspecifics (Song et al., 2023), and the PL–nucleus accumbens (NAc) oscillatory coherence increased during cooperation (Conde-Moro et al., 2024). Despite these advances, the broader cortical–subcortical networks that regulate the balance between prosocial and selfish behavior remain largely unexplored. In addition, most studies of social decision-making in mice have focused on C57BL/6 mice, an inbred strain with a well-characterized prosocial phenotype (Wu et al., 2021; Scheggia et al., 2022; Misiołek et al., 2023; Pozo et al., 2023; Song et al., 2023; Zhang et al., 2024), leaving open the question of whether these neural mechanisms generalize across genetically diverse populations.

To address these gaps, we examined neural substrates of social decision-making using inbred male C57BL/6 mice and outbred male CD1 mice. In addition to considering the regions mentioned above, namely, mPFC, NAc core (NAcC) and shell (NAcS), MD, and BLA, we also considered the lateral hypothalamus (LH), implicated in social competition (Padilla-Coreano et al., 2022); the ventral tegmental area (VTA), which encodes the motivational value of social interactions (Hung et al., 2017); and the ventral hippocampus (vHPC), which processes social memory critical for guiding social decisions (Phillips et al., 2019). Here, using a social decision-making task combined with c-Fos mapping, we examined activity in a broad network during social decision-making in both inbred and outbred mouse strains. Our findings show that increased activity in the mPFC and NAcC is associated with biases toward selfish choices in CD1 male mice. At the network level, social decision-making recruited coordinated activity between VTA, NAcC, and the mPFC. However, network dynamics in prosocial versus selfish mice differed, with distinct prefrontal–subcortical network configurations recruited that correlated with prosocial preference, with BLA as a common hub.

## Materials and Methods

### Animals

All experimental animals were maintained on a 12 h reverse light cycle (lights off, 8:30 A.M.–8:30 P.M.), and all behavioral testing was conducted during the dark phase between 9:00 A.M. and 6:00 P.M. Male CD1 (*n* = 24; Charles River Laboratories) and C57BL/6 (*n* = 24; The Jackson Laboratory) mice were 8 weeks old upon arrival and group-housed (four per cage). Behavioral experiments began at least 1 week after arrival. Mice were handled for at least 2 d prior to testing. Throughout the experiment, animals were food-restricted to 90% of their baseline body weight, with water available *ad libitum*. All procedures were performed in accordance with the University of Florida regulations and were approved by the Institutional Animal Care and Use Committee.

### Behavioral assays

#### Social decision-making task

**Figure 1.**
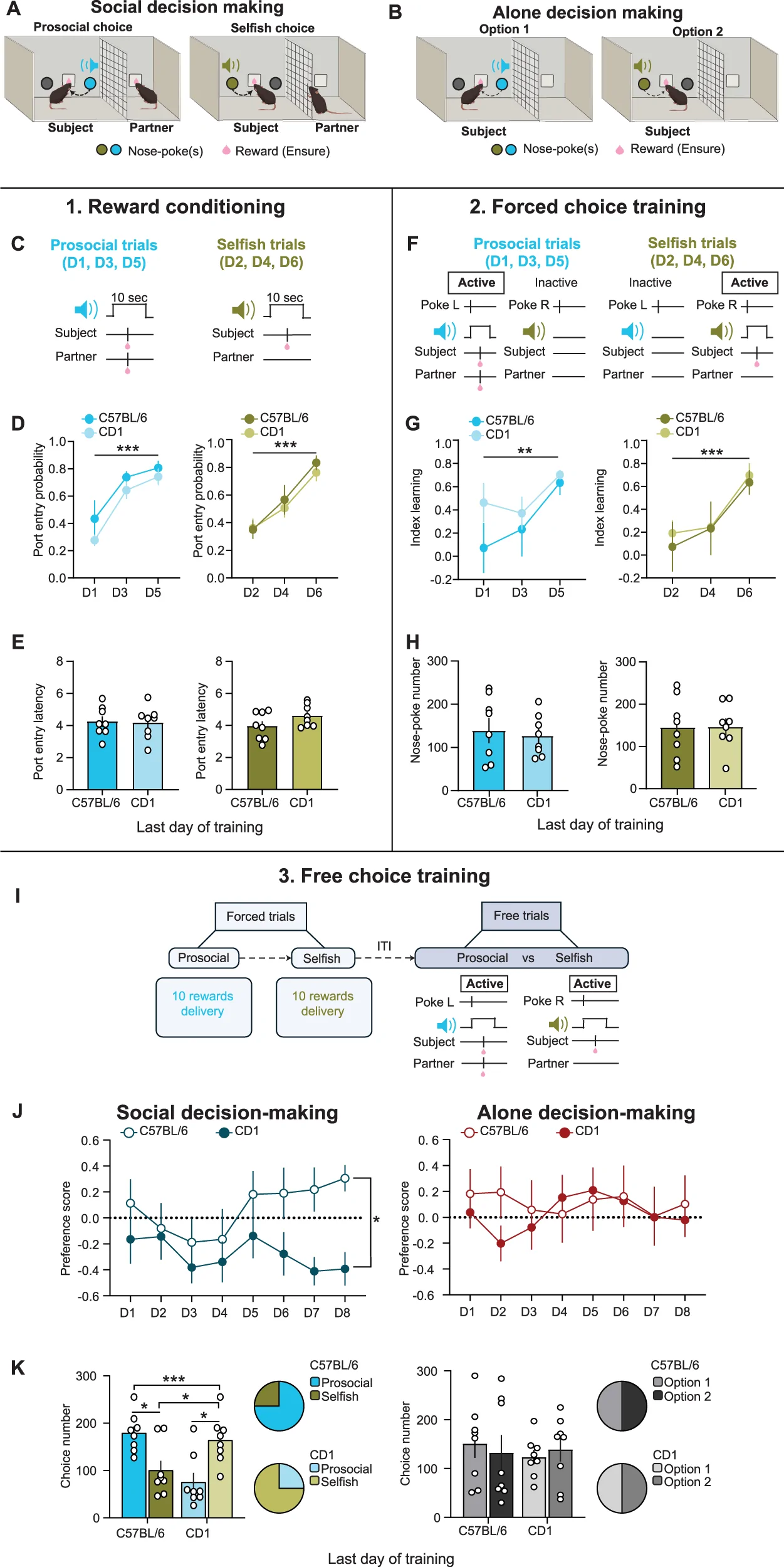
Strain differences in social decision-making. ***A***, Social decision-making task. C57/BL6 and CD1 mice (*n* = 8 per strain) choose between two nose-poke options: one that delivers a reward to themselves (selfish choice) and another that rewards them and a familiar partner (prosocial choice), who is a passive recipient (*n* = 8 per strain). ***B***, C57BL/6 (*n* = 8) and CD1 (*n* = 8) mice trained without a partner served as controls. ***C***, Reward conditioning (stage1). Subject and partner mice received two distinct tones: one paired with rewards for both mice (prosocial trials) and another paired with rewards only for the subject mice (selfish trials). Prosocial (Days 1, 3, and 5) and selfish (Days 2, 4, and 6) trials were alternated every day. ***D***, Reward port entry probability during prosocial and selfish trials in both C57BL/6 and CD1 mice across training days. ***E***, Reward port entry latencies during prosocial and selfish trials in both C57BL/6 and CD1 mice at the end of training show learning over days. ***F***, Forced-choice training. Nose-poke responses were associated with prosocial or selfish outcomes. An active nose-poke triggered the previously conditioned tones, followed by reward delivery to both mice (prosocial trials) or to the subject only (selfish trials), whereas the inactive nose-poke had no consequences. Active and inactive nose-pokes were alternated daily across sessions (prosocial, Days 1, 3, and 5; selfish, Days 2, 4, and 6). ***G***, Index learning shows discrimination between active and inactive nose-pokes during prosocial and selfish trials across days in both mouse strains. ***H***, Nose-poke responses did not differ between mouse strains during prosocial or selfish trials at the end of training. ***I***, Free-choice training. After a block of forced trials (20 reward deliveries as a criterion), mice received free-choice trials with both nose-pokes activated. ***J***, Left, Preference score (prosocial − selfish choices/ sum of both) showed higher prosocial preference in C57BL/6 mice compared with CD1 mice during social decision-making. Right, Preference score showed no side preference during alone decision-making. ***K***, Left, Choice number during the last day of training. C57BL/6 mice made more prosocial choices than selfish ones, whereas CD1 mice made more selfish than prosocial choices during social decision-making. Right, C57BL/6 and CD1 mice showed a similar number of Option 1 and Option 2 choices during alone decision-making. The pie chart showed the proportions of C57BL/6 and CD1 mice that made the majority of prosocial or selfish choices during social decision-making and during alone decision-making with Option 1 or Option 2. Data are mean ± SEM; *p < 0.05; **p < 0.01; ***p < 0.001. Additional analyses and training data from Stages 1 and 2 of the alone decision-making group are provided in Extended Data Figures 1-1 and 1-2.

C57BL/6 and CD1 mice (16 mice per strain) were trained individually in Med-PC operant chambers connected to a custom 3D-printed triangular chamber. Mice placed in the operant chambers were designated as subjects, whereas their cagemates positioned in the adjacent triangular compartment were designated as partners (Fig. 1*A*). The two compartments were separated by a metal mesh (1 cm openings) that allowed for direct social interaction. Each chamber was equipped with a modified 3D-printed reward port connected to a syringe pump (Med Associates, PHM-210), which delivered 15 µl of vanilla Ensure per reward. Each session lasted up to 40 min and was controlled using custom-written Med-PC programs. Operant chambers and partners' compartments were cleaned with soapy water and 70% of alcohol between animals.

#### Social decision-making training

All mice underwent training for the social decision-making task, which consisted of three consecutive stages: (1) reward conditioning, (2) forced-choice training, and (3) free-choice training.

1. Reward conditioning (Fig. 1*C*): Subjects and partners were confined to their respective compartments and trained to associate two distinct auditory cues, serving as conditioned stimuli (CS), with prosocial (reward delivered to both mice) or selfish (reward delivered to the subject only) outcomes. Tone–outcome pairings were counterbalanced across mice such that, for half of the animals, one tone predicted the prosocial outcome and the other predicted the selfish outcome, whereas this mapping was reversed in the remaining animals to prevent cue bias. The CS–outcome assignments established during this phase were maintained throughout subsequent training stages. Each session consisted of 30 trials of a single CS–outcome contingency (either prosocial or selfish). In each trial, the CS was presented for 10 s, and reward delivery occurred 5 s after CS onset, midway through cue presentation. Trials were separated by a variable intertrial interval (ITI) of 1–3 min. Contingencies alternated across 6 consecutive days, with 3 d of exclusive exposure to each outcome (prosocial, Days 1, 3, and 5; selfish, Days 2, 4, and 6). Learning performance was assessed by quantifying the number of reward port entries during CS presentation.
2. Forced-choice training (Fig. 1*F*): Subjects and partners were confined to their respective compartments, with the subject having access to two nose-poke ports. Mice learned to associate prosocial and selfish outcomes with their respective CS's. The assignment of prosocial and selfish contingencies to the left or right nose-poke was counterbalanced across mice and remained fixed for each animal throughout subsequent sessions and training stages. In each session, only one nose-poke was activated, whereas responses at the other port had no programmed consequences. The active nose-poke was illuminated to indicate availability, while the inactive port remained unlit. Responses at the active nose-poke triggered presentation of the associated CS (5 s duration), followed by reward delivery to both mice (prosocial) or to the subject only (selfish). The reward was delivered 2.5 s after CS onset, midway through cue presentation. Following each reinforced response, a 5 s timeout period was imposed, during which additional nose-poke responses did not trigger CS presentation or reward delivery. Prosocial and selfish sessions were alternated across 6 consecutive days, with 3 d of exclusive exposure to each outcome (prosocial, Days 1, 3, and 5; Selfish, days 2, 4, and 6). Learning performance was assessed by quantifying responses at active and inactive nose-pokes across trials.
3. Free-choice trials (Fig. 1*I*): Subjects and partners were confined to their respective compartments and first received a block of forced-choice trials, followed by 30 min of free-choice trials during which both nose-pokes were available (indicated by illumination of both ports). Forced trials were conducted as described above and terminated once each mouse had received 20 rewards for each contingency (prosocial and selfish). The order of prosocial and selfish forced-trial blocks was counterbalanced across days and across animals. At the conclusion of the forced block, a 30 s ITI preceded the onset of free-choice trials. During free-choice trials, responses at either nose-poke triggered presentation of the corresponding CS (prosocial or selfish) and reward delivery as previously described. The 5 s postreinforcement timeout described above was maintained throughout this phase. Mice were trained for 8 consecutive days until they exhibited a stable preference for one contingency for at least 3 consecutive days. Learning performance was assessed by quantifying responses at the prosocial or selfish nose-pokes.

#### Alone decision-making task

C57BL/6 and CD1 mice in the alone condition were trained using the same apparatus and trial structure as the social decision-making task, except that no partner was present (Fig. 1*B*). These animals were naive to the social setup and had no prior experience with the prosocial contingency. To preserve consistency with the social condition, nose-poke stimuli retained their original CS–side assignments and were analyzed as Option 1 and Option 2, corresponding to the two counterbalanced stimulus–side configurations established during training. Importantly, Option 1 and Option 2 did not correspond to any specific stimulus identity or spatial location; rather, they reflected arbitrary CS–side pairings that were fixed within each subject and counterbalanced across animals. Thus, in the alone condition, these labels did not imply social preference but were defined solely by stimulus identity and side assignment.

#### Social interactions

Social interaction bouts, defined as any face-to-face encounter between the subject and recipient through the metal mesh barrier lasting at least 1 s, were quantified during the c-Fos test day using manual scoring in the BORIS software by an experimenter blind to strain and experimental condition. Behaviors such as proximity without direct face orientation were not included. The total number of social interaction bouts was quantified across the entire session.

#### Social rank assays

Given that prior studies have demonstrated that social rank influences social decision-making (Gachomba et al., 2022; Scheggia et al., 2022), we controlled for this variable by measuring social ranks and using dominant mice as subjects and subordinate mice as partners in both mouse strains. To assess social rank, we used the tube test for C57BL/6 mice and the urine-marking assay for CD1 mice, based on prior evidence that these tests reliably measure dominance hierarchies in their respective strains (Cum et al., 2024).

Tube test: All training and testing were conducted in a dimly lit (20 lux), quiet room. Mice underwent 3 consecutive days of tube training prior to testing. During training, mice were guided to traverse the tube, turn at the opposite end, and return to the starting point, completing 10 full crossings per session. A plastic cylinder, slightly smaller than the tube, was used when necessary to gently prevent retreat or encourage forward movement. During testing, each mouse was paired once per session with each of its cagemates. The loser was defined as the first animal to exit the tube with all four paws, whereas the winner was defined as the animal that remained inside the tube. The order of pairings and starting positions was counterbalanced across days. Tube testing was conducted for at least 8 consecutive days and continued until stable rank hierarchies were maintained for at least 3 consecutive days.

Urine-marking test: CD1 mice were tested in a dimly lit room (20 lux) within a clear plastic enclosure (16.5 × 39.0 cm) without flooring. The enclosure was divided into two equal compartments by a clear, perforated partition that allowed visual and olfactory interaction. Chromatography paper (Whatman cellulose) was placed beneath the enclosure and covered with a metal mesh to prevent chewing. Mice were tested against different cagemates across 6 consecutive days, with sessions lasting 3 h each. Urine markings were quantified under UV illumination by trained observers. Each pair within a cage was tested in 1–2 sessions, and each mouse was tested no more than once per day. The number of urine marks per session was used to determine territorial-based social rank. Matches were considered tied when the difference between animals was <20% or when there were fewer than five urine spots.

#### Food-restriction control group

A separate cohort of C57BL/6 and CD1 mice was group-housed, food-restricted for 20 d and subsequently perfused to serve as a baseline c-Fos control group. Mice were maintained at 90% of their initial body weight and were provided with 3 g of standard chow daily at a consistent time each day. Animals were handled for at least 5 consecutive days prior to perfusion.

### Histology

Subject mice (C57BL/6: social, *n* = 8; alone *n* = 4; CD1: social, *n* = 8; alone *n* = 4) and food-restricted control mice (*n* = 8 per strain) were perfused 90 min after the final behavioral session (Day 9, Stage 4) or after an equivalent undisturbed 90 min period in their home cages, respectively. For perfusion, mice received an intraperitoneal injection of pentobarbital (31.5 mg/ml), followed by transcardial perfusion with 0.9% sodium chloride solution and 4% paraformaldehyde (PFA) in 1× phosphate-buffered saline (PBS). Brains were extracted, postfixed in 4% PFA for 24 h, and then cryoprotected in 30% sucrose in 1× PBS until the brain sank to the bottom of the tube. Brains were then sliced at 40 µm using a microtome, and slices were stored at 4°C in 0.05% sodium azide until staining. A C57B/6 brain was significantly damaged during slicing and excluded due to issues with tissue quality. 

### Immunohistochemistry

Brain sections were incubated for 1 h in a blocking solution consisting of PBS supplemented with 3% normal donkey serum (NDS) and 0.2% Triton X-100. Sections were then incubated overnight at 4°C with a rabbit monoclonal recombinant IgG primary antibody (Synaptic Systems; 1:5,000 dilution in PBS-T containing 3% NDS). The next day, sections were incubated for 2 h at room temperature in goat anti-rabbit IgG (H + L) cross-absorbed secondary antibody conjugated with Alexa Fluor 555 (Thermo Fisher Scientific; dilution 1:500 in PBS-T with 3% NDS). After washing, sections were mounted using Fluoromount-G with DAPI (Thermo Fisher Scientific). For each brain region, three sections were collected at anterior, medial, and posterior coordinates, with the sampled hemisphere counterbalanced across animals. Regions of interest were manually delineated and imaged at 20× magnification using a fluorescence microscope, ensuring consistent sampling of representative areas. Imaging parameters were kept constant across all animals and regions. c-Fos–positive cells were quantified for each section, and the mean count per region was calculated by averaging across the three sections.

### Data collection and analysis

#### Social and alone decision-making task

Learning performance was evaluated at each training stage. For Stage 1, the probability of entry into the reward port was calculated as the proportion of trials in which at least one port entry occurred within the 10 s window following CS onset (0–10 s), defined as probability entry = (number of trials with ≥1 entry)/(total number of trials). Reward port entry latency was defined as the time of the first port entry minus CS onset for that trial. Mean latency was calculated by averaging across trials with at least one entry for each subject and day. For Stages 2 and 3, the number of nose-poke responses was quantified using the MED-PC software (Med Associates). To assess discrimination between active and inactive nose-pokes, a learning index was calculated as follows: index learning = (number of active nose-pokes − number of inactive nose-pokes)/(total number of nose-pokes), where values close to 1 indicate preferential responding at the active nose-poke, values close to −1 indicate preferential responding at the inactive nose-poke, and values near 0 indicate no discrimination. To quantify individual preferences for prosocial over selfish responses, a decision preference score was calculated as follows: preference score = (number of prosocial responses − number of selfish responses)/(total number of responses), wherein values close to 1 indicate prosocial preference, values close to 0 indicate no preference, and values close to −1 indicate selfish preference. To assess side and tone preferences, the proportion of prosocial and selfish choices was calculated as follows: percentage choice = (number of prosocial or selfish responses × 100)/(total number of responses). Percentages were then grouped by side (left vs right) or auditory cue (Tone 1 vs Tone 2) and compared to assess potential biases.

#### Immunoreactivity quantification

Brain sections were imaged at 20× magnification using a KEYENCE BZ-X800 all-in-one fluorescence microscope (Keyence Corporation of America) under high-sensitivity settings. Maximum projection images were generated using the Full Focus option. Images were generated for the ACC (bregma 1.53–0.85 mm), PL (bregma 2.09–1.41 mm), IL (bregma 1.97–1.41 mm), aIC (bregma 1.53–0.85 mm), pIC (bregma 0.73–0.47 mm), NAcC (bregma 1.53–0.97 mm), NAcS (bregma 1.69–0.73 mm), MD (bregma −0.59 to −1.79 mm), BLA (bregma −1.07 to −1.79 mm), LH (bregma −0.71 to −1.79 mm), vHPC(bregma −2.79 to −3.51 mm), and VTA (−3.15 to −3.79 mm; Paxinos and Franklin's *The Mouse Brain*, 5th edition, 2019). c-Fos^+^ cells were hand-counted using the ImageJ Macro Cell Counter plugin. Positive Fos-like immunoreactivity was indicated by strong cell nuclei staining. Cell counts were performed by two experimenters blinded to experimental conditions (raters). For cell counting, each image was assigned to a single rater, with brain regions and experimental conditions counterbalanced across raters. For each animal, counts were averaged across anterior, medial, and posterior rostrocaudal levels to obtain a single mean c-Fos^+^ value per brain region.

#### Statistical analysis

**Figure 2.**
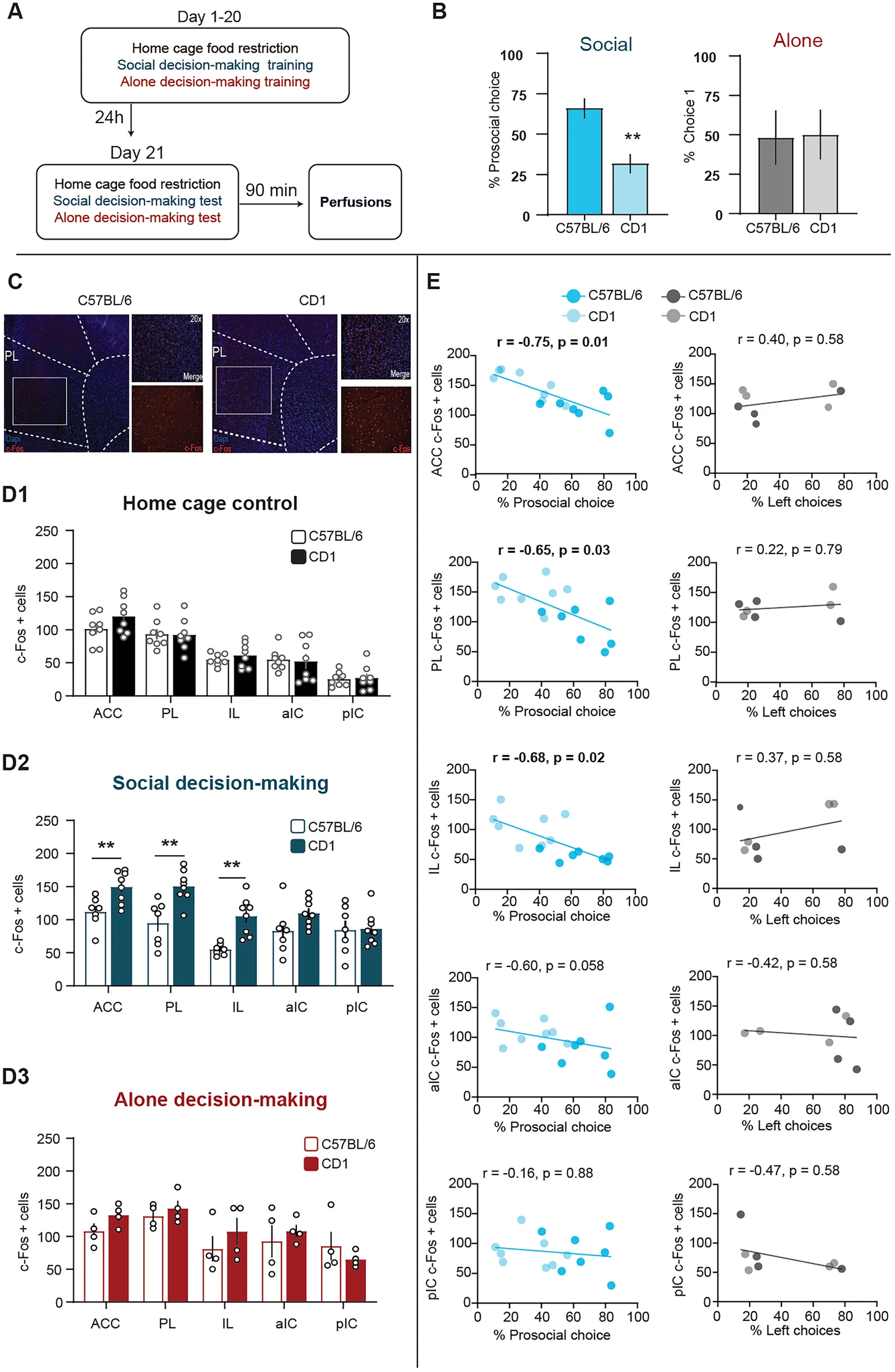
Prefrontal c-Fos activity is associated with selfish behavior. ***A***, Experimental timeline. C57BL/6 and CD1 mice were tested in one of three conditions: home cage food restriction (baseline c-Fos), social decision-making, or alone decision-making. After 21 d, mice were perfused for c-Fos mapping. ***B***, The percentage of prosocial choice during social and alone decision-making in mice prior to c-Fos mapping. ***C***, Representative c-Fos images for PL of C57BL/6 and CD1 mice during social decision-making. ***D***, Mean c-Fos–positive cells count across ACC, PL, IL, aIC, and pIC of C57 and CD1 mice under home cage (***D*1**; *n* = 8 per strain), social (***D*2**; C57BL/6 *n* = 7; CD1, *n* = 8), and alone decision-making (***D*3**; *n* = 4 per strain). ***E***, Top, Relationship between cortical subregion c-Fos and the percentage of prosocial choices during social decision-making or the percentage of left choice during alone decision-making. *P* values were adjusted using a Benjamini–Hochberg FDR procedure. Group data are presented as mean ± SEM; **p < 0.01. Additional control analyses are provided in Extended Data Figures 2-1–2-4.

**Figure 3.**
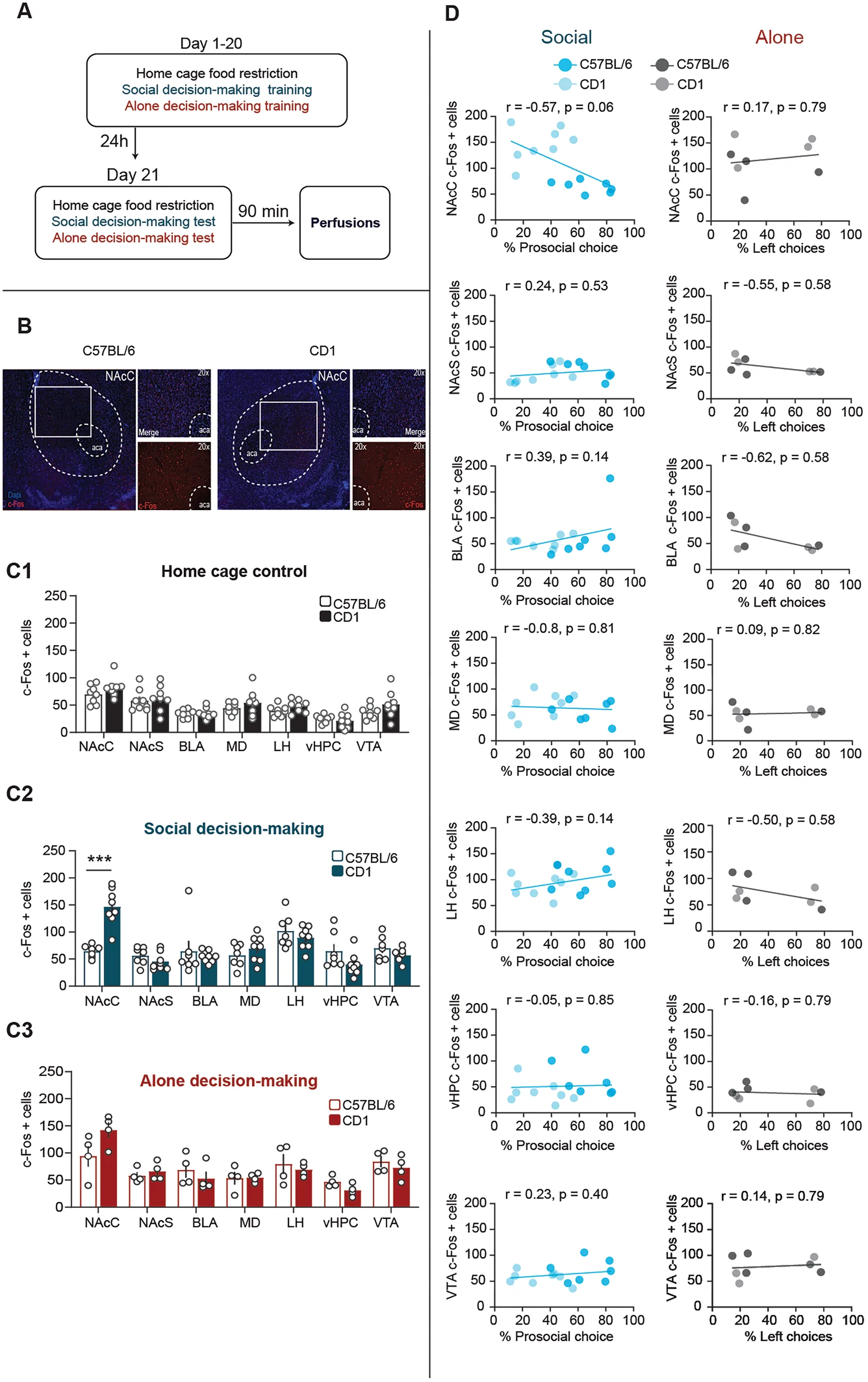
Elevated NAcC c-Fos activation in selfish CD1 mice. ***A***, Experimental timeline. ***B***, Representative c-Fos images for C57BL/6 and CD1 mice for NacC during social decision-making. ***C***, Mean c-Fos–positive cells count across NAcC, NAcS, BLA, MD, LH, vHPC, and VTA of C57 and CD1 mice under (***C*1**) home cage, (***C*2**) social, and (***C*3**) alone decision-making. ***D***, Top, Relationship between subcortical c-Fos expression and the percentage of prosocial choices during social decision-making or left choice during alone decision-making. *P* values were adjusted using a Benjamini–Hochberg FDR procedure. Group data are presented as mean ± SEM; ***p < 0.001. Additional control analyses are provided in Extended Data Figures 2-1–2-4.

Statistical analyses included Shapiro–Wilk tests for normality, Student's *t* tests, two-way and three-way ANOVAs, Pearson's correlations, and linear regression to visualize best-fit trends in scatterplots. Cell counts across brain regions were correlated with the percentage of prosocial choices (Figs. 2, 3), number of rewards consumed (Fig. S3), and number of social interactions (Fig. S4), using Pearson's test. For these behavioral correlations, *p* values were adjusted using the Benjamini–Hochberg false discovery rate (FDR) procedure.

For network analyses, we used a permutation test (scipy.stats.permutation_test). For each correlation, we generated a null distribution by randomly shuffling cell densities within regions across 2,000 permutations while calculating Pearson's correlation coefficients. For each permutation, subject labels were shuffled within a given brain region, thus maintaining the mean c-Fos values per region. The Benjamini–Hochberg FDR procedure was applied to two-tailed *p* values derived from the permutation test. For cross-regional correlations, FDR adjustments were applied to all brain region pairs within each experimental context separately (social decision-making, selfish, prosocial, no-preference, and alone). No individual region pairs survived FDR correction (*q* < 0.05). Given small sample sizes, correlations within the top and bottom 2.5% percentile of the null distribution and *p* < 0.05 in the permutation test were reported in network graphs to visualize strong cross-regional associations.

To assess coordinated network-level organization, we used a component-based permutation procedure inspired by the network-based statistic (NBS) framework (Zalesky et al., 2010). Pairwise Pearson’s correlations were computed across all selected brain regions (12 regions; 66 unique region pairs). A primary threshold of *p* < 0.01 (uncorrected) was used to define suprathreshold edges, and connected components were identified based on edge extent (number of edges). Component significance was assessed using 5,000 permutations. For each permutation, animal values were independently shuffled within each brain region (column-wise permutation, preserving missing values), thereby disrupting cross-regional dependencies while preserving the marginal distributions of each region. For each permuted dataset, the correlation matrix was recomputed, and the maximal component extent was recorded to generate a null distribution. The component-level *p* value (p_component) was defined as the proportion of permutations yielding a component extent greater than or equal to the observed extent (with a +1 correction: (*k* + 1)/(n_perm + 1). Component-based permutation tests were performed separately within each condition/subgroup to assess the presence of coordinated network organization.

To examine if coordinated activity was associated with behavioral preference, we calculated a c-Fos similarity index for each subject, focusing on each significant edge identified by the NBS analysis. The similarity index between two connected brain regions was calculated as similarity index = 1 − |*A* − *B*|/(*A* + *B*), where *A* and *B* denote the numbers of c-Fos–positive cells in each brain region per subject. This index ranges from 0 to 1, with values approaching 1 indicating more similar c-Fos activity across regions and values approaching 0 indicating greater differences in regional activity. Similarity indices were calculated separately for each selected edge and correlated with the percentage of prosocial choices across all social animals using Pearson's correlation.

## Results

### Prosocial and selfish behaviors differ between strains of male mice

This study evaluated neural activity associated with prosocial and selfish behavior at a network level in two different strains of mice (C57BL/6 and CD1). Given that past studies show that in operant-based prosocial choice assays, C57BL/6 male mice are more prosocial than females (Scheggia et al., 2022), our study focused on male mice. To study prosocial and selfish behavior, we modified a recently published task for social decision-making wherein mice decide between a prosocial option that delivers a reward to a familiar partner and a selfish option that provides a reward only to themselves (Scheggia et al., 2022). To facilitate associative learning between decisions and social outcomes, we incorporated a CS prior to operant conditioning that signals what happens to the partner and a forced decision block to ensure that subject mice sampled both options prior to freely choosing (Fig. 1).

10.1523/ENEURO.0207-26.2026.f1-1 Figure 1-1 **Learning performance of partner mice and subjects trained alone.** **A.** Top. Protocol training**.** Reward port entry probability during prosocial and selfish trials for C57BL/6 and CD1 partner mice under social decision-making (two-way RM ANOVA; Prosocial: strain, F(1,14) = 1.48, p = 0.24; days, F(1.18, 16.73) = 14.65, p = 0.0002; ; strain × day, F (2, 28) = 0.29, p = 0.74; Selfish: strain, F(1,14) = 1.81, p = 0.19, days, F(1.83, 25.64) = 0.13, p = 0.85; ; strain × day, F(2, 28) = 0.92, p = 0.40) **B.** Reward conditioning for C57BL/6 and CD1 mice trained alone. Top, protocol training. Bottom, reward port entry probability during CS-Option 1and CS-Option 2 trials showed no strain differences across days (two-way RM ANOVA; CS-Option 1 trials: strain, F(1, 14) = 3.41, p = 0.08; days, F(1.88, 26.35) = 24.59, p < 0.001; strain × day, F(2, 28) = 0.09, p = 0.91; CS-Option 2 trials: strain, F(1,14) = 3.93, p = 0.06; days, F(1.88, 26.42) = 27.25, p < 0.001; strain × day, F(2, 28) = 1.22, p = 0.30). **C.** Reward port entry latencies during CS-Option 1 and CS-Option 2 for alone-trained mice do not show strain differences during the last day of training (unpaired t-student test: prosocial, t(14) = 0.1, p = 0.84; selfish, t(14) = 0.16, p = 0.87). **D.** Forced-choice training during alone decision-making. Top, Protocol training. Bottom, Index learning indicates discrimination between active and inactive nose-pokes during CS-Option 1 and CS-Option 2 trials across strains of mice (Option 1: two-way RM ANOVA; strain, F(1, 14) = 0.35, p = 0.56; days, F(2, 28) = 6.61, p = 0.004; strain × day, F(2, 28) = 0.27, p = 0.76; Option 2: two-way RM ANOVA; strain, F(1, 14) = 0.14, p = 0.71; days, F(2, 28) = 0.86, p = 0.43; strain × day, F(2, 28) = 1.12, p = 0.34). **E.** Numbers of active vs inactive nose-poke responses on the last day of training for each trial type (unpaired t-student test: option 1, t(14) = 1.22, p = 0.24; option 2, t(14) = 0.02, p = 0.98). Download Figure 1-1, TIF file.

10.1523/ENEURO.0207-26.2026.f1-2 Figure 1-2 **No intrinsic side or tone preference during social decision-making.** **A.** Side preference during social decision-making. On the final day of free-choice training, the percentage of choices did not differ between left and right nose-pokes across mouse strains. (two-way RM ANOVA; strain, F(1, 14) = 0.00, p = 0.99; nose-poke side, F(1,14) = 0.44, p = 0.51; strain × nose-poke side, F(1,14) = 0.00, p = 0.99). **B,** Tone preference during social decision-making. On the final day of free-choice training, the percentage of choices did not differ between tones across mouse strains (two-way RM ANOVA; strain, F(1, 14) = 0.09, p = 0.75; tones, F(1,14) = 0.00, p = 0.99; strain × tone, F(1,14) = 0.97, p = 0.34). Download Figure 1-2, TIF file.

Social decision-making task training (20 d) consisted of three consecutive stages: (1) reward conditioning training (Fig. 1*C*), (2) forced-choice training (Fig. 1*F*), and (3) free-choice training (Fig. 1*I*). This protocol was designed to allow mice to express a social preference between two social outcomes after forced exploration of both options and thus facilitate associations between actions and social outcomes while minimizing side preferences formed by habit. To determine whether the presence of a partner guides choice behavior, an additional group of mice was trained under the same conditions but without a partner, serving as a control group (alone decision-making). In this condition, subjects decided between two options that delivered the same amount of reward but used the same two CS as our social group (Fig. 1*B*).

During Stage 1, reward conditioning, mice associated distinct tones with the prosocial or selfish outcomes (CS-prosocial or CS-selfish). Each CS was presented with a variable ITI 30 times per day and alternated across days (CS-prosocial Days 1, 3, and 5; CS-selfish 2, 4, and 6; Fig. 1*C*). All subject mice showed successful conditioning to both CS as demonstrated by probability for port entries increasing over days (Fig. 1*D*; two-way RM ANOVA; CS-prosocial, strain, *F*_(1,14)_ = 2.85; *p* = 0.11; days, *F*_(2,28)_ = 19.41; *p* < 0.001; strain × day, *F*_(2,28)_ = 0.21; *p* = 0.80; CS-selfish, strain, *F*_(1,14)_ = 0.25; *p* = 0.62; days, *F*_(2,28)_ = 30.22; *p* < 0.001; strain × day, *F*_(2,28)_ = 0.29; *p* = 0.74). Importantly, by the end of Stage 1, reward port entry latency did not differ by strain (Fig. 1*E*; unpaired-test, CS-prosocial, *t*_(14)_ = 0.14; *p* = 0.88; CS-selfish, *t*_(14)_ = 1.65; *p* = 0.11). Partner mice, those passively receiving rewards, also showed successful conditioning and discriminated between the CS-prosocial and CS-selfish, evidenced by increased port entries selectively to CS-prosocial (Extended Data Fig. 1-1*A*; two-way RM ANOVA; CS-prosocial, strain, *F*_(1,14)_ = 1.48; *p* = 0.24; days, *F*_(1.18,16.73)_ = 14.65; *p* = 0.0002; strain × day, *F*_(2,28)_ = 0.29; *p* = 0.74; CS-selfish, strain, *F*_(1,14)_ = 1.81; *p* = 0.19; days, *F*_(1.83,25.64)_ = 0.13; *p* = 0.85; strain × day, *F*_(2,28)_ = 0.92; *p* = 0.40). This behavioral change in partners provides important additional social cues for subjects to learn the social outcomes of their choices. Finally, mice in the alone decision-making group (Fig. 1*B*) showed successful acquisition of the CS-reward associations (Extended Data Fig. 1-1*B,C*).

In Stage 2, forced-choice training, mice were trained to associate nose-pokes with the prosocial or selfish CS and reward outcomes (Fig. 1*F*). Every day, mice were presented with one nose-poke that was active at a time, and nose-pokes elicited presentation of the CS-prosocial or the CS-selfish, followed by the corresponding reward outcomes. Using this configuration, mice were forced to use one nose-poke per session. All mice showed successful discrimination of active versus inactive nose-pokes across days, showing increased nose-pokes at the active ports (two-way RM ANOVA; prosocial, strain, *F*_(1,14)_ = 1.09; *p* = 0.31; days, *F*_(1.89,26.64)_ = 7.26; *p* = 0.003; strain × day, *F*_(2,28)_ = 1.052; *p* = 0.36; selfish, strain, *F*_(1,14)_ = 0.08; *p* = 0.7; days, *F*_(1.94,27.19)_ = 14.92; *p* < 0.001; strain × day, *F*_(2,28)_ = 0.14; *p* = 0.86). Neither strain showed nose-poke preferences in this forced stage (Fig. 1*H*; unpaired *t* test, prosocial, *t*_(14)_ = 0.66; *p* = 0.51; selfish, *t*_(14)_ = 0.10; *p* = 0.91). Mice in the alone decision-making group also discriminated between active and inactive nose-pokes across days (Extended Data Fig. 1-1*D*,*E*). These results indicate comparable discrimination between active and inactive nose-pokes across strains, demonstrating that both C57BL/6 and CD1 mice learned the operant associations.

Finally, in Stage 3, free-choice training, we measured free-choice preferences between prosocial and selfish outcomes. Each session started with counterbalanced forced trials to ensure mice sampled both options, followed by 30 min with both nose-pokes active to evaluate choice preference (Fig. 1*I*). Across days, there was a significant main effect of strain, with C57BL/6 mice displaying higher prosocial choices preference than CD1 mice (Fig. 1*J*; two-way RM ANOVA; strain, *F*_(1,14)_ = 4.66; *p* = 0.04), whereas neither the effect of days (*F*_(4.08,57.8)_ = 1.3; *p* = 0.26) nor the strain × days interaction reached significance (*F*_(7,98)_ = 1.42; *p* = 0.20). In contrast, alone decision-making resulted in no side preferences (Fig. 1*J*; two-way RM ANOVA; strain, *F*_(1,14)_ = 0.12; *p* = 0.73; days, *F*_(4.09,57.32)_ = 0.81; *p* = 0.52; strain × day, *F*_(7,98)_ = 1.057; *p* = 0.39). In the last day of social training, C57BL/6 mice showed significantly more prosocial than selfish choices, whereas CD1 mice exhibited the opposite pattern (Fig. 1*K*; two-way RM ANOVA; strain, *F*_(1,14)_ = 2.21; *p* = 0.15; nose-poke, *F*_(1,14)_ = 0.39; *p* = 0.95; strain × nose-poke, *F*_(1,14)_ = 15.08; *p* = 0.001; post hoc comparisons, prosocial choice C57BL/6 vs CD1, *p* = 0.01; selfish choice C57BL/6 vs CD1, *p* = 0.0007; C57BL/6 prosocial vs selfish, *p* = 0.02; CD1 prosocial vs selfish, *p* = 0.02). In contrast, during alone decision-making, there was no choice preference (Fig. 1*K*; two-way RM ANOVA; strain, *F*_(1,14)_ = 0.41; nose-poke, *F*_(1,14)_ = 0.001; *p* = 0.96; strain × nose-poke, *F*_(1,14)_ = 0.23; *p* = 0.63). Choice percentages did not differ across nose-poke sides or auditory cues on the final training day in either strain (two-way RM ANOVA; all *p* > 0.5), indicating that strain differences in social preference were driven by partner presence rather than task-related variables (Extended Data Fig. 1-2). Together, our results confirm previous evidence of prosocial behavior in C57BL/6 mice (Scheggia et al., 2022; Song et al., 2023) while uncovering new findings about strain differences and antisocial preferences in CD1 male mice.

### mPFC activity correlates with social choices

Given the strain differences in social decision-making, we next examined whether brain activity patterns explained differences in social decision-making. Once mice showed a stable preference for selfish or prosocial choices, C57BL/6 and CD1 mice were tested on an additional day of social or alone decision-making (Fig. 2*A*,*B*). To evaluate brain activity patterns, we labeled c-Fos expression immunohistochemically and compared the mean number of c-Fos^+^ cells in cortical regions implicated in prosocial behaviors, specifically prefrontal and insular cortices (Rogers-Carter et al., 2018; Yamagishi et al., 2020; Cox et al., 2022; Song et al., 2023; Conde-Moro et al., 2024).

Importantly, in a naive baseline home cage condition where mice were food-restricted but naive to the operant chamber, there were no strain differences in c-Fos expression in mPFC subregions (ACC, PL, and IL), anterior IC (aIC), and posterior IC (pIC; Fig. 2*D1*; two-way RM ANOVA; strain, *F*_(1,14)_ = 0.63; *p* = 0.44; regions, *F*_(2.44,36.24)_ = 48.72; *p* = <0.0001; strain × region, *F*_(4,56)_ = 0.84; *p* = 0.50). On the other hand, c-Fos expression during social decision-making differed across strains. CD1 mice had higher number of c-Fos^+^ cells in the ACC, PL, and IL compared with C57BL/6 mice (Fig. 2*D*2; two-way RM ANOVA; strain, *F*_(1,13)_ = 16.70; *p* = 0.001; regions, *F*_(2.89,37.58)_ = 13.06; *p* < 0.0001; strain × region, *F*_(4,52)_ = 2.29; *p* = 0.02; post hoc comparison, *p* = 0.01). Importantly, these differences were not observed during alone decision-making, indicating that specifically social decision-making recruited more prefrontal activity in CD1 compared with C57BL/6 mice (Fig. 2*D*3; two-way RM ANOVA; strain, *F*_(1,6)_ = 0.68; *p* = 0.44; regions, *F*_(3.02,18.12)_ = 6.32; *p* = 0.004; strain × region, *F*_(4,24)_ = 0.98; *p* = 0.43).

Given that increased prefrontal activity was not due to overall mouse strain differences, we hypothesized that these differences were due to their social choices. To test this hypothesis, we correlated the number of c-Fos^+^ cells with the percentage of prosocial choices (Fig. 2*E*). c-Fos activity was anticorrelated with the percentage of prosocial choices in ACC (*r* = −0.75; *p* = 0.014), PL (*r* = −0.65; *p* = 0.03), and IL (*r* = −0.68; *p* = 0.02). Because animals tested in the alone condition had no real prosocial option, we instead examined whether the percentage of left nose-poke choices (Fig. 2*E*) and Tone 1 choices (Extended Data Fig. 2-1) correlated with c-Fos expression to rule out a side or auditory preference. No significant correlations were observed in the alone condition, indicating that the correlations observed in the social decision-making group are specific to the social context rather than driven by stimulus or side preferences.

10.1523/ENEURO.0207-26.2026.f2-1 Figure 2-1 **Tone-guided choices do not correlate with c-Fos activity in the alone condition.** Cortical and subcortical c-Fos–positive cell counts as a function of the percentage of tone 1 choices during alone decision-making across all mice. Download Figure 2-1, TIF file.

### Strain-specific increases in NAcC c-Fos activity during social decision-making

We next examined c-Fos levels in select subcortical regions associated with social behaviors (Hung et al., 2017; Phillips et al., 2019; Padilla-Coreano et al., 2022; Scheggia et al., 2022; Conde-Moro et al., 2024) across strains in a naive baseline home cage group, as well as social and alone decision-making groups. After baseline home cage food-restriction condition, there was no significant effect of strain in c-Fos^+^ cells in subcortical regions (Fig. 3*C*1; two-way RM ANOVA; strain, *F*_(1,14)_ = 2.15; *p* = 0.16; regions, *F*_(3.96,55.45)_ = 22.48; *p* < 0.0001; strain × region, *F*_(6,84)_ = 0.82; *p* = 0.55). However, c-Fos activity during social decision-making showed a significant interaction between strain and region, with higher NAcC c-Fos expression in CD1 mice (Fig. 3*C*2; two-way RM ANOVA; strain, *F*_(1,13)_ = 0.44; *p* = 0.51; regions, *F*_(3.70,41.21)_ = 11.12; *p* < 0.0001; strain × region, *F*_(6,78)_ = 7.94; *p* < 0.0001; post hoc comparison, *p* = 0.001). On the other hand, c-Fos activity during alone decision-making also showed a significant interaction between strain and region, yet no specific region had significant differences between strains (Fig. 3*C*3; two-way RM ANOVA; strain, *F*_(1,6)_ = 0.002; *p* = 0.95; regions, *F*_(2.84,17.09)_ = 11.83; *p* < 0.0002; strain × region, *F*_(6,36)_ = 2.42; *p* = 0.04; NAcC, post hoc comparison, *p* = 0.09). These results suggest that NAcC activity could be driven by more selfish choices in CD1 mice. NAcC c-Fos^+^ cells and the percentage of prosocial choices showed a trending anticorrelation (Fig. 3*D*), suggesting that social choices may influence c-Fos expression in NAcC during social decision-making in CD1 mice.

Importantly, when considering all groups together, a significant effect of context (baseline home cage, social, and alone decision-making) on c-Fos expression was observed across both cortical and subcortical regions. For subcortical regions, a significant effect was found for the region–context–strain interactions (three-way RM ANOVA, *F*_(12,231)_ = 2.5035; *p* = 0.0042). For cortical regions, although there was no significant three-way interaction, all pairs of effects had significant interactions (three-way RM ANOVA; region × strain, *F*_(4,165)_ = 2.634; *p* = 0.0360; region × condition, *F*_(8,165)_ = 2.276; *p* = 0.0246; strain × condition, *F*_(2,165)_ = 6.9385; *p* = 0.00128). These findings indicate that different brain regions are differentially engaged depending on the behavioral context and strain, suggesting that c-Fos expression differences across strains are driven by context-induced behavioral differences rather than purely genetic differences.

Given that mPFC activity and NAcC are associated with reward processing (Burgos-Robles et al., 2013; Soares-Cunha et al., 2020) and social interactions (Lee et al., 2016; Dai et al., 2022), we evaluated whether c-Fos activity during social decision-making was influenced by reward and social interactions. No significant correlations were observed between c-Fos expression and the total number of rewards consumed during social decision-making (Extended Data Fig. 2-2*A*) nor the alone decision-making (Extended Data Fig. 2-2*B*). Despite significant differences in the number of face-to-face social interactions across strains of mice (Extended Data Fig. 2-3*A*), the total number of interaction bouts did not correlate with either the percentage of prosocial choices (Extended Data Fig. 2-3*B*) or c-Fos expression in cortical or subcortical brain regions (Extended Data Fig. 2-3*C*), indicating that c-Fos activity was not related to general reward consumption or social engagement. Additionally, c-Fos expression in the social condition was not correlated with side choices (percentage of left nose-poke responses) or tone-guided choices (percentage of Tone 1 responses; Extended Data Fig. 2-4), indicating that these activity patterns are not explained by spatial or auditory biases. Overall, these results reinforce that these activity patterns are driven by differences in social choices.

10.1523/ENEURO.0207-26.2026.f2-2 Figure 2-2 **Number of rewards obtained does not correlate with c-Fos activity.** **A.** Cortical and subcortical c-Fos–positive cell counts as a function of rewards consumed during social decision-making across all mice. **B.** Cortical and subcortical c-Fos–positive cell counts as a function of rewards consumed during alone decision-making across all mice. Download Figure 2-2, TIF file.

10.1523/ENEURO.0207-26.2026.f2-3 Figure 2-3 **Increased social interaction in C57BL/6 mice is not associated with c-Fos activity. A.** Number of social interactions during the c-Fos test in C57BL/6 and CD1 mice (unpaired t-test, t(13) = 2.46, p = 0.02). **B.** Correlation between the number of social interactions and the percentage of prosocial choices. **C.** Cortical and subcortical c-Fos + cells count as a function of the number of social interactions during social decision-making across all mice. Download Figure 2-3, TIF file.

**Figure 4.**
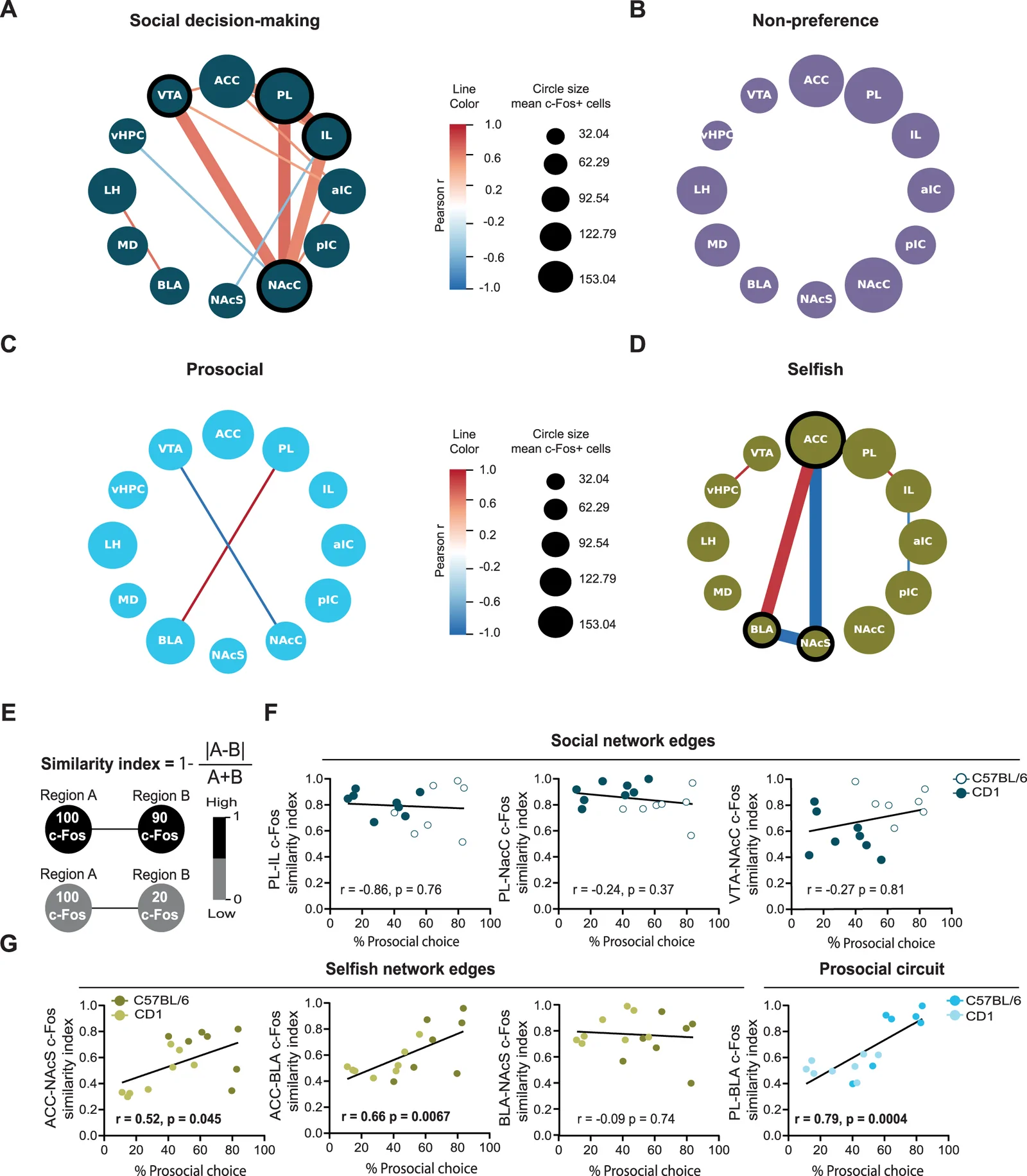
Cross-regional c-Fos correlations across social decision-making conditions. ***A–D***, Networks showing significant cross-correlations based on a null-distribution method during social decision-making for all mice (***A***), no-preference mice (***B***), prosocial mice (***C***), and selfish mice (***D***). Line color represents the Pearson’s *r* value. Only cross-correlations in the top/bottom 2.5% of the null distribution and *p* < 0.05 in the permutation test are shown. Black circle outlines and thick lines indicate components identified as significant by the NBS (*p* < 0.01). The circle size represents the mean c-Fos expression for each region. Sample sizes for social decision-making (*n* = 7 C57 and 8 CD1), prosocial mice (*n* = 5 C57), selfish (*n* = 1 C57 and 5 CD1), and non-preference (*n* = 1 C57 and 3 CD1). ***E***, Schematic representation of the c-Fos similarity index, calculated as 1−(∣A−B∣/A+B), where ***A*** and ***B*** represent the numbers of c-Fos–positive cells in two connected brain regions. Values approaching 1 indicate more similar c-Fos activity. ***F, G***, Correlation between the c-Fos similarity index and the percentage of prosocial choices for significant edges from the social selfish subnetworks identified by NBS and the prosocial PL–BLA circuit. Complete correlation and NBS analyses for all groups are provided in Extended Data Figure 4-1.

10.1523/ENEURO.0207-26.2026.f2-4 Figure 2-4 **Side- and tone-guided choices do not correlate with c-Fos activity during social decision-making. A**. Cortical and subcortical c-Fos–positive cell counts as a function of the percentage of left nose-poke choices during social conditions across all mice. **B.** Cortical and subcortical c-Fos–positive cell counts as a function of the percentage of tone 1 choices during social decision-making across all mice. Download Figure 2-4, TIF file.

### Network states and cross-regional correlations depend on social decisions

To investigate how interactions across regions relate to social decision-making, we next examined functional connectivity among these cortical and subcortical regions. Because baseline c-Fos expression did not differ between C57BL/6 and CD1 mice (Figs. 2*D*, 3*C*), and to assess networks recruited for general social decision-making, data from both strains was combined for cross-correlation analyses of c-Fos^+^ cells across brain regions. Functional connectivity was assessed first by using NBS (5,000 permutations; primary threshold *p* < 0.01; Zalesky et al., 2010), as social decision-making is thought to engage distributed circuits rather than isolated regional interactions (Rogers-Carter and Christianson, 2019). Because NBS identifies significant connected subnetworks rather than individual pairwise interactions, we additionally examined individual pairwise relationships. No individual pairwise edges remained significant after FDR correction for multiple comparisons (Extended Data Fig. 4-1*A*–*E*); therefore, pairwise correlations are presented as descriptive measures of connectivity, whereas statistical inference is based on the NBS analysis. Pairwise cross-correlations of c-Fos^+^ cell counts between brain region pairs were assessed using permutation testing, as previously described (De Paula Cunha Almeida et al., 2025).

During social decision-making, a significant connected subnetwork component (extent, three edges; *p* = 0.0068) comprising the VTA, NAcC, PL, and IL was found (Fig. 4*A*; Table 1). These results indicate coordinated subnetwork activity within a corticostriatal–midbrain subnetwork during social decision-making, supporting functional coupling among prefrontal (PL, IL), striatal (NAcC), and midbrain (VTA) regions. This was further supported by pairwise positive correlations that exceeded chance levels between mPFC subregions, NAcC, and VTA (PL–IL, *r* = 0.6790; *p* = 0.0029; *q* = 0.0989; percentile = 99.9; NAcC–PL, *r* = 0.7065; *p* = 0.0019; *q* = 0.0989; percentile = 99.9; NAcC–IL, *r* = 0.6022; *p* = 0.0229; *q* = 0.2088; percentile = 98.9; VTA–NAcC, *r* = 0.6523; *p* = 0.0089; *q* = 0.1979; percentile = 99.6). Importantly, none of these cross-correlations were significant in the alone decision-making group, and NBS found no significant subnetworks in this group (Extended Data Fig. 4-1*F*; Table 2). We also observed more pairwise correlations that exceeded chance levels in social decision-making (13 pairwise relationships; Fig. 4*A*; Extended Data Fig. 4-1*A*; Table 1) than in the alone condition (three pairs; Extended Data Fig. 4-1*E*,*F*; Table 2). Altogether, these findings suggest that coordinated corticostriatal–midbrain coupling is specifically recruited to enable social decision-making.

10.1523/ENEURO.0207-26.2026.f4-1 Figure 4-1 **Cross-regional c-Fos correlations during alone decision-making.** Heatmaps showing pairwise Pearson correlations of c-Fos + cell counts across brain regions during social decision-making for all mice (**A**), no preference mice (**B**), prosocial mice (**C**), selfish mice (**D**), and alone mice (**E**). **F**. Network visualizations show significant cross-correlations for the alone condition based on a null-distribution method. Line color represents Pearson r value. Only cross-correlations in the top/bottom 2.5% of null distribution are shown (n = 4 C57 and 4 CD1). Social (n = 7 C57 and 8 CD1), non-preference (n = 1 C57 and 3 CD1), prosocial (n = 5 C57), selfish (n = 1 C57 and 5 CD1). Download Figure 4-1, TIF file.

**Table 1.** Pearson's correlations of mean c-Fos^+^ cells count across brain regions during social decision-making

Pair region	Pearson's R	Permutation *p* value	Adjusted *p* value (FDR)	Percentile of observed value in the null distribution	*n*
**ACC–PL**	**0.581949898**	**0.01999001**	**0.208895552**	**99.05**	**15**
**ACC–IL**	**0.543343631**	**0.03798101**	**0.208895552**	**98.15**	**15**
**ACC–aIC**	**0.556415301**	**0.03598201**	**0.208895552**	**98.25**	**15**
ACC–pIC	0.352968271	0.19990005	0.527736132	90.05	15
ACC–NAcC	0.474069113	0.05997002	0.247376312	97.05	15
ACC–NAcS	−0.51456661	0.05477551	0.254485657	2.65	15
ACC–BLA	−0.02334483	0.97651174	0.976511744	48.8	15
ACC–MD	0.373511449	0.17491254	0.501922952	91.3	15
ACC–LH	0.002540277	0.94452774	0.974044228	52.8	15
ACC–vHPC	−0.158109	0.54772614	0.83958021	27.35	15
**ACC–VTA**	**0.586379709**	**0.0129935**	**0.208895552**	**99.4**	**15**
**PL–IL**	**0.67885679**	**0.0029985**	**0.098950525**	**99.9**	**15**
PL–aIC	0.50856697	0.05097451	0.228685657	97.5	15
PL–pIC	0.156826789	0.55772114	0.83958021	72.15	15
**PL–NAcC**	**0.706528415**	**0.001999**	**0.098950525**	**99.95**	**15**
PL–NAcS	−0.1669304	0.53573213	0.83958021	26.75	15
PL–BLA	0.135544302	0.64567716	0.887806097	67.75	15
PL–MD	0.32212734	0.22388806	0.568331219	88.85	15
PL–LH	0.086148421	0.7876062	0.928250161	60.65	15
PL–vHPC	−0.42340361	0.12793603	0.425487256	6.35	15
PL–VTA	0.406713747	0.12893553	0.425487256	93.6	15
IL–aIC	0.297373859	0.28485757	0.66605407	85.8	15
IL–pIC	−0.19299457	0.50874563	0.839430285	25.4	15
**IL–NAcC**	**0.602268296**	**0.02298851**	**0.208895552**	**98.9**	**15**
**IL–NAcS**	**−0.54227003**	**0.03298351**	**0.208895552**	**1.6**	**15**
IL–BLA	−0.13995899	0.7836082	0.928250161	39.15	15
IL–MD	0.028243553	0.91554223	0.960186573	54.25	15
IL–LH	0.04337691	0.83858071	0.954247014	58.1	15
IL–vHPC	−0.15067607	0.62568716	0.887806097	31.25	15
IL–VTA	0.406096984	0.14992504	0.471192975	92.55	15
aIC–pIC	0.415085022	0.12093953	0.425487256	94	15
**aIC–NAcC**	**0.588739836**	**0.01999001**	**0.208895552**	**99.05**	**15**
aIC–NAcS	−0.11291904	0.71564218	0.926125173	35.75	15
aIC–BLA	0.069387369	0.75362319	0.928250161	62.35	15
aIC–MD	0.178885951	0.50674663	0.839430285	74.7	15
aIC–LH	−0.19275765	0.44377811	0.770772508	22.15	15
aIC–vHPC	−0.24298591	0.3948026	0.743466105	19.7	15
aIC–VTA	0.511406291	0.05197401	0.228685657	97.45	15
pIC–NAcC	−0.01489024	0.97251374	0.976511744	48.6	15
pIC–NAcS	−0.0505559	0.83458271	0.954247014	41.7	15
pIC–BLA	0.230284807	0.4067966	0.743466105	79.7	15
pIC–MD	0.356219862	0.18990505	0.522238881	90.55	15
pIC–LH	−0.03332337	0.91654173	0.960186573	45.8	15
pIC–vHPC	0.03615425	0.87056472	0.960186573	56.5	15
pIC–VTA	0.044077866	0.90654673	0.960186573	54.7	15
NAcC–NAcS	−0.13961707	0.58770615	0.861969015	29.35	15
NAcC–BLA	−0.18815136	0.69265367	0.926125173	34.6	15
NAcC–MD	0.249804227	0.35682159	0.727636182	82.2	15
NAcC–LH	−0.08298228	0.7856072	0.928250161	39.25	15
**NAcC–vHPC**	**−0.52682354**	**0.03698151**	**0.208895552**	**1.8**	**15**
**NAcC–VTA**	**0.652379075**	**0.00899550**	**0.197901049**	**99.6**	**15**
NAcS–BLA	−0.1777702	0.64367816	0.887806097	32.15	15
NAcS–MD	−0.16574212	0.55972014	0.83958021	27.95	15
NAcS–LH	−0.32949241	0.24387806	0.596146371	12.15	15
NAcS–vHPC	0.281692252	0.31284358	0.66605407	84.4	15
NAcS–VTA	−0.22922223	0.4167916	0.743466105	20.8	15
BLA–MD	0.12571286	0.71464268	0.926125173	64.3	15
**BLA–LH**	**0.674444304**	**0.03198401**	**0.208895552**	**98.45**	**15**
BLA–vHPC	−0.21103092	0.4007996	0.743466105	20	15
BLA–VTA	−0.33123579	0.16191904	0.485757121	8.05	15
MD–LH	0.291701801	0.30684658	0.66605407	84.7	15
MD–vHPC	−0.40706297	0.12593703	0.425487256	6.25	15
MD–VTA	0.030620477	0.90054973	0.960186573	55	15
LH–vHPC	−0.27440574	0.31084458	0.66605407	15.5	15
LH–VTA	−0.24351178	0.36381809	0.727636182	18.15	15
vHPC–VTA	−0.08886915	0.7846077	0.928250161	39.2	15

Adjusted *p* values were obtained from permutation-derived null distributions of Pearson’s correlation coefficients and corrected for multiple comparisons using the Benjamini–Hochberg FDR. Percentile values indicate the position of each observed correlation relative to the permutation-based null distribution. Bold rows indicate correlations falling within the upper or lower 2.5% of the null distribution and with permutation *p* < 0.05 (uncorrected). *n* indicates the number of animals included in each correlation analysis.

**Table 2.** Pearson's correlations of mean c-Fos^+^ cells count across brain regions during alone decision-making

Pair region	Pearson's *R*	Permutation *p* value	Adjusted *p* value (FDR)	Percentile of observed value in the null distribution	*n*
ACC–PL	0.045413914	0.943528236	0.988458	52.85	8
ACC–IL	0.242573136	0.60169915	0.891154	69.95	8
ACC–aIC	−0.01156472	0.990504748	0.990505	49.5	8
ACC–pIC	−0.1318678	0.785607196	0.925894	39.25	8
ACC–NAcC	0.450295652	0.286856572	0.891154	85.7	8
ACC–NAcS	0.224724944	0.604697651	0.891154	69.8	8
ACC–BLA	−0.27964017	0.498750625	0.891154	24.9	8
ACC–MD	0.350436814	0.410794603	0.891154	79.5	8
ACC–LH	−0.40156532	0.335832084	0.891154	16.75	8
ACC–vHPC	−0.25568887	0.526736632	0.891154	26.3	8
ACC–VTA	−0.33991422	0.396801599	0.891154	19.8	8
PL–IL	0.631301432	0.124937531	0.775112	93.8	8
PL–aIC	0.505515368	0.187906047	0.775112	90.65	8
PL–pIC	0.078234353	0.831584208	0.944782	58.45	8
PL–NAcC	0.527294147	0.177911044	0.775112	91.15	8
PL–NAcS	−0.51636538	0.15992004	0.775112	7.95	8
PL–BLA	−0.04758013	0.971514243	0.990505	48.55	8
PL–MD	−0.1886315	0.585707146	0.891154	29.25	8
PL–LH	0.59586216	0.151924038	0.775112	92.45	8
PL–vHPC	−0.00643244	0.978510745	0.990505	48.9	8
PL–VTA	0.706407119	0.045977011	0.775112	97.75	8
IL–aIC	0.252035803	0.544727636	0.891154	72.8	8
IL–pIC	0.378668657	0.52173913	0.891154	73.95	8
IL–NAcC	0.446561097	0.261869065	0.891154	86.95	8
IL–NAcS	−0.35822793	0.374812594	0.891154	18.7	8
IL–BLA	−0.11464297	0.724637681	0.891154	36.2	8
IL–MD	0.576001068	0.111944028	0.775112	94.45	8
IL–LH	0.171764934	0.667666167	0.891154	66.65	8
IL–vHPC	−0.33396607	0.446776612	0.891154	22.3	8
IL–VTA	0.416277593	0.289855072	0.891154	85.55	8
aIC–pIC	0.382505567	0.357821089	0.891154	82.15	8
aIC–NAcC	0.545496544	0.182908546	0.775112	90.9	8
aIC–NAcS	−0.18113222	0.707646177	0.891154	35.35	8
aIC–BLA	0.505056795	0.178910545	0.775112	91.1	8
aIC–MD	−0.13190477	0.84057971	0.944782	42	8
**aIC–LH**	**0.801177812**	**0.011994003**	**0.775112**	**99.45**	**8**
aIC–vHPC	−0.38207164	0.354822589	0.891154	17.65	8
aIC–VTA	0.32199103	0.468765617	0.891154	76.6	8
pIC–NAcC	0.110848154	0.844577711	0.944782	57.8	8
pIC–NAcS	0.056631978	0.741629185	0.891154	62.95	8
**pIC–BLA**	**0.727138841**	**0.030984508**	**0.775112**	**98.5**	**8**
pIC–MD	0.63362717	0.056971514	0.775112	97.2	8
pIC–LH	0.531188246	0.263868066	0.891154	86.85	8
pIC–vHPC	0.127789943	0.736631684	0.891154	63.2	8
pIC–VTA	0.387476549	0.429785107	0.891154	78.55	8
NAcC–NAcS	−0.10763719	0.742628686	0.891154	37.1	8
NAcC–BLA	0.31615185	0.485757121	0.891154	75.75	8
NAcC–MD	0.12444853	0.700649675	0.891154	65	8
NAcC–LH	0.227709009	0.603698151	0.891154	69.85	8
NAcC–vHPC	−0.56363248	0.150924538	0.775112	7.5	8
NAcC–VTA	0.360059097	0.394802599	0.891154	80.3	8
NAcS–BLA	0.107428751	0.734632684	0.891154	63.3	8
NAcS–MD	0.194671269	0.616691654	0.891154	69.2	8
NAcS–LH	−0.32403511	0.435782109	0.891154	21.75	8
NAcS–vHPC	0.0512625	0.898550725	0.96397	55.1	8
NAcS–VTA	−0.67659103	0.086956522	0.775112	4.3	8
BLA–MD	0.165057426	0.72163918	0.891154	63.95	8
BLA–LH	0.590067421	0.134932534	0.775112	93.3	8
BLA–vHPC	0.01857546	0.897551224	0.96397	55.15	8
BLA–VTA	0.404743882	0.295852074	0.891154	85.25	8
MD–LH	−0.24515489	0.540729635	0.891154	27	8
MD–vHPC	−0.20480099	0.614692654	0.891154	30.7	8
MD–VTA	−0.06720861	0.905547226	0.96397	45.25	8
LH–vHPC	0.159517564	0.68165917	0.891154	65.95	8
LH–VTA	0.657736234	0.071964018	0.775112	96.45	8
vHPC–VTA	0.220192255	0.595702149	0.891154	70.25	8

Adjusted *p* values were obtained from permutation-derived null distributions of Pearson’s correlation coefficients and corrected for multiple comparisons using the Benjamini–Hochberg FDR. Percentile values indicate the position of each observed correlation relative to the permutation-based null distribution. Bold rows indicate correlations falling within the upper or lower 2.5% of the null distribution and with permutation *p* < 0.05 (uncorrected). *n* indicates the number of animals included in each correlation analysis.

We next evaluated whether social choices influenced the network state. We separated our subject population according to their overall preferred social choice on the last day of training and grouped them into prosocial, selfish, and nonpreference mice. Mice with no social preference had no significant pairwise correlation nor subnetwork activity (Fig. 4*B*; Table 3). On the other hand, mice with clear social preferences showed distinct network interactions. Prosocial mice had two pairwise correlations exceeding the chance level with PL–BLA showing a positive correlation (Fig. 4*C*; Extended Data Fig. 4-1*C*; Table 4, *r* = 0.9895; *p* = 0.0289; *q* = 0.9301; percentile = 98.9) and NAcC–VTA showing a negative correlation (Fig. 4*C*; Extended Data Fig. 4-1*C*; Table 4; *r* = −0.9326; *p* = 0.0359; *q* = 0.9301; percentile = 1.75). In contrast, selfish mice exhibited a significant connected component (NBS; extent, two edges; *p* = 0.041) comprising ACC, NAcS, and BLA (Fig. 4*D*; Extended Data Fig. 4-1*D*; Table 5), indicating that network interactions are altered depending on social preferences. Consistent with this subnetwork, the permutation test identified a correlation between ACC–BLA and anticorrelations between ACC–NAcS and BLA–NAcS that exceeded chance although were not significant after multiple-comparison corrections (ACC–BLA, *r* = 0.8980; *p* = 0.046; *q* = 0.4837; percentile = 98.25; ACC–NAcS, *r* = −0.9720; *p* = 0.049; *q* = 0.4837; percentile = 2.45; BLA–NAcS, *r* = −0.9496; *p* = 0.009; *q* = 0.4837; percentile = 0.45; Fig. 4*D*; Table 5). These results identify a novel ACC–NAcS–BLA subnetwork associated with the selfish behavioral phenotype, while the PL–BLA correlation observed in prosocial mice aligns with previous studies implicating this circuit in prosocial behavior. However, these behavioral phenotypes were strongly stratified by strain (selfish, 5 CD1, 1 C57; prosocial, 0 CD1, 5 C57).

**Table 3.** Pearson's correlations of mean c-Fos^+^ cells count across brain regions during no-preference choices

Pair region	Pearson's *R*	Permutation *p* value	Adjusted *p* value (FDR)	Percentile of observed value in the null distribution	*n*
ACC–PL	0.325951	0.666667	0.862745	68.83681	4
ACC–IL	−0.04693	0.833333	0.932203	41.05903	4
ACC–aIC	0.704478	0.25	0.862745	89.67014	4
ACC–pIC	−0.30012	0.75	0.868421	35.50347	4
ACC–NAcC	0.618089	0.333333	0.862745	84.80903	4
ACC–NAcS	0.545785	0.416667	0.862745	81.77083	4
ACC–BLA	0.214917	0.75	0.868421	66.05903	4
ACC–MD	−0.70034	0.25	0.862745	11.02431	4
ACC–LH	−0.94845	0.166667	0.862745	6.336806	4
ACC–vHPC	−0.26876	0.833333	0.932203	40.45139	4
ACC–VTA	0.818669	0.25	0.862745	89.67014	4
PL–IL	0.871141	0.166667	0.862745	93.83681	4
PL–aIC	0.845755	0.333333	0.862745	86.19792	4
PL–pIC	0.321987	0.75	0.868421	64.84375	4
PL–NAcC	0.805425	0.333333	0.862745	86.19792	4
PL–NAcS	−0.61063	0.5	0.862745	22.30903	4
PL–BLA	0.280425	0.75	0.868421	66.05903	4
PL–MD	0.430932	0.666667	0.862745	69.01042	4
PL–LH	−0.55582	0.5	0.862745	23.00347	4
PL–vHPC	−0.9981	0.083333	0.862745	1.649306	4
PL–VTA	0.484472	0.5	0.862745	76.47569	4
IL–aIC	0.674806	0.5	0.862745	77.17014	4
IL–pIC	0.721221	0.25	0.862745	89.67014	4
IL–NAcC	0.727773	0.5	0.862745	77.17014	4
IL–NAcS	−0.83531	0.25	0.862745	10.32986	4
IL–BLA	0.526076	0.416667	0.862745	81.33681	4
IL–MD	0.634098	0.416667	0.862745	81.42361	4
IL–LH	−0.2621	0.75	0.868421	34.80903	4
IL–vHPC	−0.88526	0.166667	0.862745	6.25	4
IL–VTA	−0.00377	0.916667	0.991803	56.33681	4
aIC–pIC	0.31522	0.666667	0.862745	69.01042	4
aIC–NAcC	0.980508	0.083333	0.862745	98.00347	4
aIC–NAcS	−0.18164	1	1	47.30903	4
aIC–BLA	0.563656	0.5	0.862745	78.55903	4
aIC–MD	−0.08184	1	1	51.30208	4
aIC–LH	−0.88876	0.083333	0.862745	1.475694	4
aIC–vHPC	−0.81278	0.333333	0.862745	15.10417	4
aIC–VTA	0.578917	0.666667	0.862745	69.53125	4
pIC–NAcC	0.48492	0.5	0.862745	77.95139	4
pIC–NAcS	−0.6019	0.5	0.862745	21.26736	4
pIC–BLA	0.837137	0.083333	0.862745	97.56944	4
pIC–MD	0.390935	0.583333	0.862745	73.17708	4
pIC–LH	0.016906	0.916667	0.991803	43.22917	4
pIC–vHPC	−0.33434	0.666667	0.862745	31.33681	4
pIC–VTA	−0.58764	0.416667	0.862745	19.44444	4
NAcC–NAcS	−0.23225	1	1	49.21875	4
NAcC–BLA	0.710609	0.333333	0.862745	85.50347	4
NAcC–MD	−0.05735	1	1	52.08333	4
NAcC–LH	−0.83532	0.083333	0.862745	2.170139	4
NAcC–vHPC	−0.77451	0.333333	0.862745	13.71528	4
NAcC–VTA	0.41207	0.666667	0.862745	68.83681	4
NAcS–BLA	−0.14459	0.666667	0.862745	32.55208	4
NAcS–MD	−0.95158	0.083333	0.862745	0.954861	4
NAcS–LH	−0.28559	0.666667	0.862745	30.55556	4
NAcS–vHPC	0.655404	0.416667	0.862745	82.20486	4
NAcS–VTA	0.297018	0.666667	0.862745	69.61806	4
BLA–MD	−0.14053	1	1	48.17708	4
BLA–LH	−0.43297	0.5	0.862745	23.00347	4
BLA–vHPC	−0.2578	0.75	0.868421	35.41667	4
BLA–VTA	−0.27313	0.583333	0.862745	25.78125	4
MD–LH	0.509769	0.583333	0.862745	73.17708	4
MD–vHPC	−0.48562	0.583333	0.862745	25.95486	4
MD–VTA	−0.33251	0.666667	0.862745	30.46875	4
LH–vHPC	0.503491	0.583333	0.862745	74.56597	4
LH–VTA	−0.74188	0.333333	0.862745	14.67014	4
vHPC–VTA	−0.44962	0.583333	0.862745	27.77778	4

Adjusted *p* values were obtained from permutation-derived null distributions of Pearson’s correlation coefficients and corrected for multiple comparisons using the Benjamini–Hochberg FDR. Percentile values indicate the position of each observed correlation relative to the permutation-based null distribution. *n* indicates the number of animals included in each correlation analysis.

**Table 4.** Pearson's correlations of mean c-Fos^+^ cells count across brain regions during prosocial choices

Pair region	Pearson's *R*	Permutation *p* value	Adjusted *p* value (FDR)	Percentile of observed value in the null distribution	*n*
ACC–PL	0.217658368	0.765617191	0.951524238	61.975	5
ACC–IL	−0.526373214	0.412793603	0.951524238	20.35	5
ACC–aIC	0.622969217	0.258870565	0.930134933	87.45	5
ACC–pIC	0.784899211	0.133933033	0.930134933	93.925	5
ACC–NAcC	0.182471184	0.731634183	0.951524238	63.525	5
ACC–NAcS	−0.413265333	0.493753123	0.951524238	24.35	5
ACC–BLA	0.247121923	0.767616192	0.951524238	61.925	5
ACC–MD	0.853160479	0.112943528	0.930134933	94.575	5
ACC–LH	0.497250921	0.413793103	0.951524238	79.775	5
ACC–vHPC	−0.033737416	0.897551224	0.951524238	44.425	5
ACC–VTA	−0.168907338	0.735632184	0.951524238	36.05	5
PL–IL	−0.300049216	0.665667166	0.951524238	33.05	5
PL–aIC	0.628650398	0.217891054	0.930134933	89.6	5
PL–pIC	0.771619621	0.11994003	0.930134933	94.85	5
PL–NAcC	0.076396199	0.895552224	0.951524238	55.55	5
PL–NAcS	0.438407344	0.355822089	0.951524238	82.525	5
**PL–BLA**	**0.989544784**	**0.028985507**	**0.930134933**	**98.9**	**5**
PL–MD	0.105627473	0.886556722	0.951524238	56.05	5
PL–LH	0.092701066	0.922538731	0.951524238	54	5
PL–vHPC	−0.438052608	0.48075962	0.951524238	23.625	5
PL–VTA	0.153923708	0.746626687	0.951524238	63.05	5
IL–aIC	0.003861716	0.951524238	0.951524238	47.425	5
IL–pIC	−0.472389275	0.456771614	0.951524238	22.7	5
IL–NAcC	−0.137802062	0.806596702	0.951524238	39.925	5
IL–NAcS	0.705188303	0.164917541	0.930134933	91.95	5
IL–BLA	−0.428673697	0.417791104	0.951524238	20.125	5
IL–MD	−0.67100633	0.212893553	0.930134933	10.425	5
IL–LH	−0.801038674	0.106946527	0.930134933	4.95	5
IL–vHPC	0.685732989	0.270864568	0.930134933	86.775	5
IL–VTA	0.314313987	0.55972014	0.951524238	72.3	5
aIC–pIC	0.831692914	0.143928036	0.930134933	93.4	5
aIC–NAcC	−0.18383916	0.707646177	0.951524238	35.075	5
aIC–NAcS	0.333159795	0.643678161	0.951524238	68.45	5
aIC–BLA	0.566996931	0.335832084	0.951524238	83.825	5
aIC–MD	0.470739654	0.414792604	0.951524238	79.6	5
aIC–LH	0.135052814	0.822588706	0.951524238	58.975	5
aIC–vHPC	0.286444216	0.778610695	0.951524238	61.4	5
aIC–VTA	0.431211157	0.477761119	0.951524238	76.775	5
pIC–NAcC	0.177112497	0.814592704	0.951524238	59.8	5
pIC–NAcS	0.060829456	0.950524738	0.951524238	52.825	5
pIC–BLA	0.7742662	0.144927536	0.930134933	93.7	5
pIC–MD	0.593221715	0.28185907	0.930134933	86.525	5
pIC–LH	0.326980514	0.597701149	0.951524238	70.65	5
pIC–vHPC	−0.256728747	0.547726137	0.951524238	26.925	5
pIC–VTA	−0.002955884	0.945527236	0.951524238	47.15	5
NAcC–NAcS	0.080672206	0.942528736	0.951524238	53.4	5
NAcC–BLA	0.083001672	0.932533733	0.951524238	53.875	5
NAcC–MD	−0.255638015	0.665667166	0.951524238	33	5
NAcC–LH	−0.441497681	0.447776112	0.951524238	21.825	5
NAcC–vHPC	−0.596376926	0.242878561	0.930134933	11.825	5
**NAcC–VTA**	**−0.932579986**	**0.035982009**	**0.930134933**	**1.725**	**5**
NAcS–BLA	0.316109184	0.523738131	0.951524238	74.125	5
NAcS–MD	−0.67338154	0.214892554	0.930134933	10.525	5
NAcS–LH	−0.776408966	0.127936032	0.930134933	6.175	5
NAcS–vHPC	0.183810014	0.779610195	0.951524238	61.35	5
NAcS–VTA	0.247419861	0.707646177	0.951524238	65.225	5
BLA–MD	0.174174741	0.815592204	0.951524238	59.65	5
BLA–LH	0.201851282	0.767616192	0.951524238	62.05	5
BLA–vHPC	−0.528162035	0.266866567	0.930134933	12.825	5
BLA–VTA	0.104784394	0.84157921	0.951524238	58.875	5
MD–LH	0.856849933	0.091954023	0.930134933	95.7	5
MD–vHPC	0.042745194	0.906546727	0.951524238	55.35	5
MD–VTA	0.139766096	0.778610695	0.951524238	61.5	5
LH–vHPC	−0.155284283	0.825587206	0.951524238	41.2	5
LH–VTA	0.225305631	0.693653173	0.951524238	65.35	5
vHPC–VTA	0.648735427	0.251874063	0.930134933	87.725	5

Adjusted *p* values were obtained from permutation-derived null distributions of Pearson’s correlation coefficients and corrected for multiple comparisons using the Benjamini–Hochberg FDR. Percentile values indicate the position of each observed correlation relative to the permutation-based null distribution. Bold rows indicate correlations falling within the upper or lower 2.5% of the null distribution and with permutation *p* < 0.05 (uncorrected). *n* indicates the number of animals included in each correlation analysis.

**Table 5.** Pearson's correlations of mean c-Fos^+^ cells count across brain regions during selfish choices

Pair region	Pearson's *R*	Permutation *p* value	Adjusted *p* value (FDR)	Percentile of observed value in the null distribution	*n*
ACC–PL	0.79899858	0.08395802	0.54972514	95.95	6
ACC–IL	0.63919977	0.16091954	0.65307346	92	6
ACC–aIC	−0.02242893	0.95252374	0.98229011	47.6	6
ACC–pIC	−0.33718806	0.49075462	0.83087028	24.5	6
ACC–NAcC	0.24229948	0.73463268	0.91865179	63.3	6
**ACC–NAcS**	**−0.97196454**	**0.04997501**	**0.48375812**	**2.45**	**6**
**ACC–BLA**	**0.89795043**	**0.04697651**	**0.48375812**	**97.775**	**6**
ACC–MD	0.20792106	0.74862569	0.91865179	62.65	6
ACC–LH	0.38969145	0.46976512	0.83087028	76.625	6
ACC–vHPC	−0.42502641	0.27686157	0.67616192	13.7	6
ACC–VTA	−0.35497302	0.52773613	0.83087028	26.125	6
**PL–IL**	**0.88323757**	**0.03998001**	**0.48375812**	**98.15**	**6**
PL–aIC	−0.1580957	0.73763118	0.91865179	36.85	6
PL–pIC	−0.51575715	0.28685657	0.67616192	14.175	6
PL–NAcC	0.41835825	0.44977511	0.82458771	77.625	6
PL–NAcS	−0.80481633	0.09495252	0.54972514	4.7	6
PL–BLA	0.79695568	0.06596702	0.48375812	96.8	6
PL–MD	−0.27125813	0.65167416	0.90842079	32.4	6
PL–LH	0.76203483	0.09995003	0.54972514	95.15	6
PL–vHPC	−0.10347592	0.83858071	0.97677477	41.75	6
PL–VTA	−0.04773977	0.91154423	0.98229011	45.55	6
IL–aIC	−0.04487563	0.92053973	0.98229011	46	6
**IL–pIC**	**−0.8538831**	**0.02298851**	**0.48375812**	**0.95**	**6**
IL–NAcC	0.29153014	0.57471264	0.88211708	71.425	6
IL–NAcS	−0.65287574	0.13393303	0.63139859	6.6	6
IL–BLA	0.78379388	0.06096952	0.48375812	97.15	6
IL–MD	−0.60757415	0.18990505	0.65307346	9.15	6
IL–LH	0.60430281	0.26386807	0.67616192	87	6
IL–vHPC	0.01919351	0.94852574	0.98229011	52.75	6
IL–VTA	0.2365015	0.66066967	0.90842079	67.25	6
aIC–pIC	−0.16804033	0.75162419	0.91865179	37.55	6
aIC–NAcC	0.54936167	0.27786107	0.67616192	86.225	6
aIC–NAcS	−0.17737503	0.59870065	0.88982176	29.8	6
aIC–BLA	0.31238282	0.52873563	0.83087028	73.675	6
aIC–MD	−0.05984923	0.99550225	0.99550225	50.3	6
aIC–LH	0.0385885	0.98750625	0.99550225	50.65	6
aIC–vHPC	−0.81046554	0.05397301	0.48375812	2.625	6
aIC–VTA	−0.60510964	0.22388806	0.67166417	11.075	6
pIC–NAcC	−0.08493723	0.89655172	0.98229011	44.775	6
pIC–NAcS	0.36871098	0.44277861	0.82458771	78.125	6
pIC–BLA	−0.6136692	0.19790105	0.65307346	9.775	6
pIC–MD	0.76292528	0.11494253	0.58355438	94.45	6
pIC–LH	−0.27305794	0.60669665	0.88982176	30.225	6
pIC–vHPC	−0.05872924	0.94852574	0.98229011	47.4	6
pIC–VTA	−0.41168201	0.3838081	0.76961519	19.1	6
NAcC–NAcS	−0.38795024	0.3848076	0.76961519	19.15	6
NAcC–BLA	0.44085138	0.36381809	0.76961519	81.95	6
NAcC–MD	−0.20284433	0.72463768	0.91865179	36.1	6
NAcC–LH	0.47390981	0.35382309	0.76961519	82.45	6
NAcC–vHPC	−0.61545268	0.16991504	0.65307346	8.425	6
NAcC–VTA	−0.60805919	0.18490755	0.65307346	8.95	6
NAcS–BLA	−0.94962218	0.009995	0.48375812	0.425	6
NAcS–MD	−0.15706609	0.84357821	0.97677477	42	6
NAcS–LH	−0.50193311	0.35082459	0.76961519	17.45	6
NAcS–vHPC	0.56669996	0.15492254	0.65307346	92.45	6
NAcS–VTA	0.46756746	0.28185907	0.67616192	86.05	6
BLA–MD	−0.11042287	0.73463268	0.91865179	36.35	6
BLA–LH	0.47393718	0.4077961	0.7916042	79.875	6
BLA–vHPC	−0.55141657	0.26786607	0.67616192	13.25	6
BLA–VTA	−0.3450541	0.50074963	0.83087028	25	6
MD–LH	−0.28260397	0.63568216	0.90842079	31.55	6
MD–vHPC	−0.37129924	0.51074463	0.83087028	25.5	6
MD–VTA	−0.60055308	0.20789605	0.65338759	10.1	6
LH–vHPC	−0.10148299	0.85857072	0.97699426	42.85	6
LH–VTA	−0.11437668	0.8125937	0.97511244	40.1	6
**vHPC–VTA**	**0.91774047**	**0.04197901**	**0.48375812**	**98.25**	**6**

Adjusted *p* values were obtained from permutation-derived null distributions of Pearson’s correlation coefficients and corrected for multiple comparisons using the Benjamini–Hochberg FDR. Percentile values indicate the position of each observed correlation relative to the permutation-based null distribution. Bold rows indicate correlations falling within the upper or lower 2.5% of the null distribution and with permutation *p* < 0.05 (uncorrected). *n* indicates the number of animals included in each correlation analysis.

Because strain strongly dictates individual subjects' social preferences, we reasoned that subnetwork differences identified through categorical comparisons might partly reflect strain rather than social preferences. Given that our baseline home cage condition showed no differences in c-Fos activity across strains (Extended Data Fig. 2-3), it remains possible that the relationship between subnetwork coordination and behavior is continuous rather than strain-specific. To test this, we asked whether c-Fos similarity within phenotype-associated subnetworks identified through NBS (Fig. 4*E*) tracked variation in prosocial preference across all subjects, independent of categorical phenotype or strain grouping. We found a significant correlation between c-Fos similarity (see Materials and Methods) and prosocial preference for two edges in the selfish subnetworks (ACC–NAcS *r* = 0.52; *p* = 0.045; ACC–BLA *r* = 0.66; *p* = 0.0067; Fig. 4*G*), whereas no such relationship was observed for edges in the general social network (PL–IL *r* = −0.86, *p* = 0.76; PL–NAcC *r* = −0.24; *p* = 0.37; VTA–NAcC *r* = −0.27; *p* = 0.81; Fig. 4*F*). This follows because the general social subnetwork was designed to capture differences in network recruitment between social and alone decision-making, regardless of behavioral phenotype, whereas the selfish subnetwork was designed to capture phenotype-specific network interactions. Given previous work implicating the PL–BLA circuit in prosocial behavior, we next investigated c-Fos similarity in this circuit and found a significant correlation with prosocial preference in all mice (PL–BLA *r* = 0.79; *p* = 0.0004; Fig. 4*G*). These findings suggest that coordinated activity within phenotype-associated subnetworks varies continuously with behavioral preference, consistent with the behavioral differences observed between strains.

## Discussion

Here, we reveal strain-dependent differences in social decision-making: CD1 mice favored selfish choices, whereas C57BL/6 mice were more prosocial. CD1 mice showed higher mPFC and NAcC c-Fos expression, with mPFC activity correlating with selfish behavior. Network analyses identified a coordinated PL–IL–NAcC–VTA subnetwork during social decision-making, suggesting that distinct prefrontal–striatal–midbrain circuits are engaged by different social strategies.

We adapted a validated social decision-making task (Scheggia et al., 2022) by adding pavlovian cue conditioning and forced sampling to ensure balanced option experience before free choice. This design aligns with established decision-making frameworks (Simon et al., 2009; St Onge and Floresco, 2009) and facilitates future electrophysiological studies through cue-aligned analyses. Combined with c-Fos mapping, it enabled assessment of distributed cortical–subcortical activity underlying socially biased decisions.

### Selfish and prosocial behavior are strain-dependent

Recent studies have established mice as a robust model for investigating prosocial behaviors (Gachomba et al., 2024). In particular, C57BL/6 mice display robust prosocial behaviors, including consolation and affiliative behaviors toward distressed (Wu et al., 2021; Zhang et al., 2024), helping trapped cagemates (Pozo et al., 2023), avoiding harm to others (Song et al., 2023), and sharing rewards with partners (Scheggia et al., 2022; Misiołek et al., 2023). Consistent with these findings, dominant C57BL/6 mice displayed a robust prosocial phenotype in our modified social decision-making paradigm. In contrast, CD1 mice exhibited a pronounced bias toward selfish choices.

Several factors may contribute to this divergence. First, social interaction is a key determinant of prosocial expression in mice (Scheggia et al., 2022; Song et al., 2023). Here, C57BL/6 mice engaged in more social interaction with their partners than CD1 mice. This further supports strain differences in baseline prosociability, as CD1 mice interacted less and were less likely to provide rewards to partner mice. Second, CD1 mice display higher levels of agonistic behaviors, including aggressive interactions, than other strains (Van Loo et al., 2003; Hsieh et al., 2017; Cum et al., 2024). Agonistic behaviors indicate social dominance and hierarchical status in mice (Zhou et al., 2018; Fulenwider et al., 2022). Consistent with this framework, CD1 mice exhibit elevated dominance-associated behaviors during social competition and increased territorial marking compared with C57BL/6 mice (Cum et al., 2024). Given that dominant mice were used as subjects in social decision-making, the selfish bias observed in CD1 mice may reflect dominance-related tendencies that prioritize individual reward even in the absence of competition. Future studies should determine whether subordinate CD1 mice display different social decision-making profiles. Finally, motivational differences may further bias social choices. CD1 mice exert greater effort to obtain rewards than C57BL/6 mice (Campos-Ordoñez and Buriticá, 2024), indicating heightened reward sensitivity. This greater valuation of individual benefits could amplify competitive tendencies, fostering selfish decisions in CD1 mice.

Taken together, these findings indicate that selfish and prosocial behaviors are not uniform features of murine social decision-making but instead vary across strains. While dominance or motivational traits were not directly evaluated here, strain-dependent behavioral tendencies reported in the literature may explain these differences during social decision-making. Such behavioral divergence provides a useful framework for interpreting the strain-specific neural activity patterns observed across cortical–subcortical circuits.

### Selective mPFC engagement underlies selfish choice bias

The mPFC is a hub for integrating decision-making (Gangopadhyay et al., 2021), and its subregions have been implicated in prosocial behavior (Song et al., 2023; Conde-Moro et al., 2024). Our data, however, show that CD1 mice exhibit higher c-Fos activation in these mPFC subregions than C57BL/6 mice and that mPFC activity is inversely correlated with prosocial choice. Thus, mPFC engagement appears preferentially linked to selfish decisions, contrary to previous reports. Notably, most prosocial studies in mice use C57BL/6 mice; thus, findings may be unintentionally biased toward phenotypically prosocial breeds.

In humans, the mPFC plays a central role in integrating self-interest during social decision-making. Functional neuroimaging studies demonstrate that mPFC activity increases with the level of self-risk during altruistic choice, indicating enhanced processing of self-related costs when individuals consider acting in their own interest (Hu et al., 2017). These findings suggest that mPFC does not exclusively promote prosocial behavior but rather encodes decision variables related to self-interest within a social context. Importantly, self-interest in social contexts often unfolds within hierarchical environments, where individuals must balance personal gain against social consequences (Sapolsky, 2005). Consistent with this broader role, medial prefrontal regions that are functionally conserved across species are critically involved in regulating social hierarchy and dominance-related behaviors (Wang et al., 2011). In rodents, activity within these subregions influences competitive interactions and hierarchical status. For instance, optogenetic activation of PL drives competitive dominance (Zhou et al., 2018), whereas ACC and PL activity are implicated when mice successfully compete for rewards (Li et al., 2022; Padilla-Coreano et al., 2022), highlighting mPFC contributions to social dominance across different competitive contexts. Given that our subject mice were of dominant rank, the elevated c-Fos expression observed in ACC, PL, and IL during selfish choice in CD1 mice may reflect recruitment of medial prefrontal circuits integrating self-interest with competitive social interactions. Together, our findings suggest that increased mPFC activation may reflect the implementation of a dominance-aligned, resource-maximizing strategy within a social context.

Although IC is implicated in prosocial behavior (Rogers-Carter et al., 2018; Cox et al., 2022), we found no significant IC recruitment during either prosocial or selfish choices. IC activity is associated with emotional contagion and affective processing in social contexts of distress (Rogers-Carter and Christianson, 2019; Keysers et al., 2022). Because our task did not present overt vulnerability or distress, the IC may have remained largely unengaged. Overall, these results highlight that strain-dependent social strategies are linked to engagement of the mPFC during social decision-making.

### NAcC is engaged in CD1 selfish mice more than C57BL/6 prosocial mice

The NAc has been related to social behaviors, including cooperative interactions (Ben-Ami Bartal et al., 2021; Conde-Moro et al., 2024; Hazani et al., 2025). In our study, however, c-Fos expression was markedly higher in the NAcC of selfish CD1 mice than in C57BL/6 mice, indicating that this region is more engaged during self-oriented decision-making. This strain-dependent activity pattern may be related to previously described differences in social dominance traits. Prior work shows that inhibiting the NAc reduces social dominance (Hollis et al., 2015; Shan et al., 2022), indicating its role in competitive behavior. Accordingly, elevated NAcC activity during selfish choices in CD1 mice may reflect striatal engagement in competitive reward acquisition rather than prosocial valuation.

Strain differences were not observed across the other subcortical regions evaluated, NAcS, BLA, MD, LH, vHPC, and VTA. Although these areas have been implicated in prosocial and dominance behaviors (Hung et al., 2017; Phillips et al., 2019; Padilla-Coreano et al., 2022; Scheggia et al., 2022; Song et al., 2023), we detected no significant c-Fos differences during social choice. Rather than ruling out their role, this suggests that social decisions depend more on coordinated circuit interactions than on isolated regional activation. Indeed, the absence of strain-level differences in these regions is consistent with their participation in the broader social decision-making network identified here, where their functional relevance may be expressed through coordinated activity with other regions rather than through differential recruitment by strain.

### Network-level organization of social decision-making circuits

Network-level analysis revealed coordinated activity across mPFC, striatal, and midbrain regions. Rather than reflecting isolated subregional effects, social decision-making was associated with subnetwork interactions among PL, IL, NAcC, and VTA. Although individual pairwise correlations did not survive multiple-comparison correction, the correlation values are strong, and a permutation test supports functionally meaningful coupling across these regions. This pattern is consistent with extensive evidence in rodents showing that corticostriatal and mesolimbic circuits coordinate reward-based and goal-directed decision-making processes (Lammel et al., 2014; Floresco, 2015; Khani and Rainer, 2016). Importantly, corticostriatal and mesolimbic circuits have been shown to regulate socially motivated behaviors, including prosocial and competitive interactions (Xing et al., 2021; Choi et al., 2024; Conde-Moro et al., 2024), suggesting that social choices may emerge from the recruitment of canonical value-based decision networks in social contexts.

Network-based statistical analyses and permutation tests of pairwise interactions suggest that prosocial and selfish mice exhibit distinct patterns of subnetwork interactions. Prosocial mice exhibited coordinated PL–BLA activity patterns consistent with previous findings (Scheggia et al., 2022). Notably, we observed a negative correlation between VTA and NAcC. While this finding does not establish directionality or mechanism, it raises the possibility that social decision-making involves dynamic regulatory interactions within this circuit, potentially reflecting VTA GABAergic inhibition over NAc during reward-seeking in stress conditions (Lowes et al., 2021). In selfish mice, negative associations between ACC–NAcS and NAcS–BLA emerged as part of a significant coordinated subnetwork. Although ACC–NAcS has not yet been explored in social behaviors, ACC to NAc projections have been implicated in social transfer of pain (Smith et al., 2021), a form of emotional contagion essential for prosocial behavior in rodents (Scheggia et al., 2022). Within this framework, the observed negative association between ACC and NAcS may reflect reduced coordination among circuits supporting emotional contagion during selfish choices. Similarly, a prior work showed increased BLA–NAc coherence during cooperative behavior (Conde-Moro et al., 2024), whereas we observed an inverse pattern in selfish mice, suggesting distinct modes of amygdala–striatal engagement during social decisions. Finally, no-preference mice showed no significant pairwise correlations nor subnetwork activity, suggesting that coordinated network activity is specifically associated with the expression of social decision preferences.

To complement these subgroup-based network analyses, we examined whether the similarity of c-Fos activity between connected regions was associated with behavioral preference across all mice independent of strain. Notably, significant relationships were observed for edges within the selfish subnetwork and the PL–BLA circuit, whereas edges in the general social network were not associated with behavioral preference. These findings suggest that coordinated activity within phenotype-associated subnetworks varies continuously with behavioral preference, providing complementary support for the subgroup-based network analyses. Nevertheless, the strain-dependent distribution of behavioral phenotypes should be considered when interpreting these relationships.

### Limitations of the study and future directions

Several limitations should be considered. First, c-Fos mapping provides indirect, temporally limited measures of neuronal activation, thereby precluding conclusions about synaptic directionality or causal interactions. Second, phenotypic and strain subgroup analyses were conducted with relatively small sample sizes, and these associations lay the groundwork for further investigation to confirm network states based on social choices. Future experiments combining circuit manipulations and in vivo multisite electrophysiological recordings will be necessary to determine the causal contributions and temporal dynamics of the identified corticostriatal–midbrain circuits during social decision-making. Third, the present study was conducted exclusively in male mice. Previous studies have reported sex differences in rodent prosocial behavior, although the direction of these differences has varied across experimental paradigms and mouse strains, when behavioral paradigms vary suggesting context-based sex differences (Scheggia et al., 2022; Misiołek et al., 2023). Future studies including female subjects will be important to determine the generalizability of these findings across sexes. Finally, the present study focused on a reward-based social decision-making paradigm. Whether the identified subnetworks are similarly engaged during other forms of prosocial behavior or social choices remains an important question for future investigation.

## Conclusion

Our findings reveal strain-dependent differences in social decision-making, with CD1 mice exhibiting greater selfish behavior, accompanied by elevated mPFC and NAcC activity. At the network level, social decision-making engaged coordinated VTA–NAc–prefrontal interactions, while prosocial and selfish mice recruited distinct cortical–subcortical configurations. Together, these results provide evidence that social decision-making is supported by distributed cortical and subcortical network dynamics that differentially shape selfish and prosocial choices.

## Synthesis

Reviewing Editor: Sam Golden, The University of Washington Decisions are customarily a result of the Reviewing Editor and the peer reviewers coming together and discussing their recommendations until a consensus is reached. When revisions are invited, a fact-based synthesis statement explaining their decision and outlining what is needed to prepare a revision will be listed below. The following reviewer(s) agreed to reveal their identity: NONE. Note: If this manuscript was transferred from JNeurosci and a decision was made to accept the manuscript without peer review, a synthesis may not be available.

Dear Dr. Padilla-Coreano,

Thank you for transferring your manuscript to eNeuro. I have assessed your rebuttal letter and revised manuscript from the JN submission, and do not believe it requires further reviewer comments. Your current version is appropriate for publication in eNeuro.
